# Schizophrenia-associated glycoprotein Adamtsl3 regulates perineuronal net formation, maintenance, and adult cortical plasticity

**DOI:** 10.1038/s41380-026-03672-7

**Published:** 2026-06-11

**Authors:** Teresa M. L. Cramer, Saad B. Hannan, Lucie Petre, Mark F. Bear, Takao K. Hensch, Shiva K. Tyagarajan

**Affiliations:** 1 University of Zurich, Institute of Pharmacology and Toxicology, Winterthurerstrasse 190, Zürich, 8057 Switzerland; 2 Department of Molecular Cellular Biology, Center for Brain Science, Harvard University, 52 Oxford Street, Cambridge, MA 02138 USA; 3 Picower Institute for Learning and Memory, Department of Brain and Cognitive Sciences, Massachusetts Institute of Technology, Cambridge, MA USA; 4 FM Kirby Neurobiology Center, Boston Children’s Hospital, Harvard Medical School, Boston, MA 02115 USA; 5 International Research Center for Neurointelligence, University of Tokyo Institutes for Advanced Study, Bunkyo-ku, Tokyo 113 Japan; 6 Present Address: University of Coimbra, Dept. of Life Sciences, Center for Neuroscience and Cell Biology, Center for Innovative Biomedicine and Biotechnology, Rua Larga - POLO I, Coimbra, 3004-504 Portugal

**Keywords:** Schizophrenia, Neuroscience

## Abstract

Perineuronal nets (PNNs) regulate the maturation of parvalbumin-positive (PV+) interneurons during postnatal critical periods and continue to support their function thereafter. Their disruption is implicated in neuropsychiatric disorders such as schizophrenia, yet key endogenous PNN regulators remain unknown. Here, we identify the schizophrenia-associated glycoprotein Adamtsl3 as a PV+ cell-autonomous regulator of PNN integrity. Using mouse genetics, morphological and biochemical analyses, we demonstrate that *Adamtsl3* deletion, both in early postnatal and adult stages, results in PNN deficits. Mechanistically, Adamtsl3 modulates matrix metalloprotease-9 (MMP9) activity, and *Adamtsl3* deletion results in elevated MMP9 levels, PNN reduction, decreased Otx2 uptake, and heightened oxidative stress in PV+ cells. MMP9 hyperactivity and PNN reduction can be rescued by pharmacological inhibition of MMP9. Deletion of *Adamtsl3* in PV+ interneurons revealed its cell-autonomous function in modulating PNN integrity. Notably, conditional *Adamtsl3* deletion in adult PV+ cells reactivated juvenile-like ocular dominance plasticity. These results position Adamtsl3 as a persistent, PV+ specific PNN regulator with direct implications for the pathophysiology of schizophrenia.

## Introduction

Perineuronal nets (PNNs) are specialized extracellular matrix (ECM) structures that primarily enwrap parvalbumin-expressing (PV+) interneurons, a major subtype of GABAergic cells in the neocortex [1]. Composed of chondroitin sulfate proteoglycans (CSPGs) such as aggrecan, versican, brevican, and neurocan [2], PNNs form dense, lattice-like sheaths around the somato-dendritic compartments of particular PV+ cells. PNN formation coincides with the maturation of PV+ cells during windows of heightened plasticity in early postnatal development called critical periods (CPs) [1, 3]. Once established, PNNs restrict experience-dependent plasticity, stabilize inhibitory networks, and maintain excitatory/inhibitory (E/I) balance [3, 4]. Growing evidence points to PNNs as key regulators of circuit architecture, and their disruption has been linked to several neurodevelopmental and neurodegenerative disorders, including schizophrenia, Alzheimer’s disease, and Fragile X Syndrome [5–10]. Defining the molecular pathways that govern PNN assembly and stability is therefore crucial for understanding both normal brain function and disease mechanisms.

Matrix metalloproteases (MMPs) and disintegrin and metalloproteinases with thrombospondin motifs (ADAMTSs) are endogenous regulators of the ECM / PNN. The ADAMTS superfamily comprises seven ADAMTS-like proteins (ADAMTSL1–6) and papilin [11, 12]. All ADAMTSL proteins are secreted glycoproteins that localize to the ECM, but their physiological functions remain poorly defined [13]. Notably, a putative functional variant in the *Adamtsl3* gene has been implicated in schizophrenia by genome-wide association studies [14, 15], yet its role in the ECM/ PNN remains unclear.

MMP9 is one of the most studied regulators of PNNs. In the primary visual cortex (V1), MMP9 expression and activity are developmentally regulated, peaking before PNN formation (prior to postnatal day 14, P14) and declining during PNN maturation (P21–P42) [16]. Juvenile *MMP9* knockout mice display precocious PNN formation [7], highlighting MMP9’s critical role in modulating PNN assembly and maintenance. MMP9 activity is tightly controlled by tissue inhibitor of metalloproteinases-1 (TIMP-1), which binds both its pro- and active forms to modulate enzymatic function in response to neuronal activity [17, 18]. Other inhibitors, including additional TIMPs and thrombospondins, further contribute to the localized regulation of MMP9, forming an intricate network that shapes PNN structure [19].

We previously identified Adamtsl3 as a critical regulator of GABAergic synapse organization in the hippocampus [20]. In the present study, we extend this work to examine its role at the PNN in V1, where PNN development and function are well characterized [1, 21]. Our findings reveal that Adamtsl3 is localized to PNNs and plays a critical role in PNN formation and adult maintenance. Mechanistically, we demonstrate that Adamtsl3 inhibits MMP9 activity. *Adamtsl3* deletion increases MMP9 activity and reduces PNNs, leading to reduced Otx2 uptake and oxidative stress in PV+ cells, which are restored upon pharmacological MMP9 inhibition. Constitutive and conditional deletion of Adamtsl3 in PV+ cells demonstrates a cell-autonomous role, with removal in adulthood reinstating juvenile-like ocular dominance plasticity. Together, these results establish Adamtsl3 as a key PV+ cell–autonomous regulator of MMP9 activity, PNN integrity, and cortical plasticity.

## Results

### Adamtsl3 localization to PNNs is crucial for their maturation and maintenance in V1

**Fig. 1:**
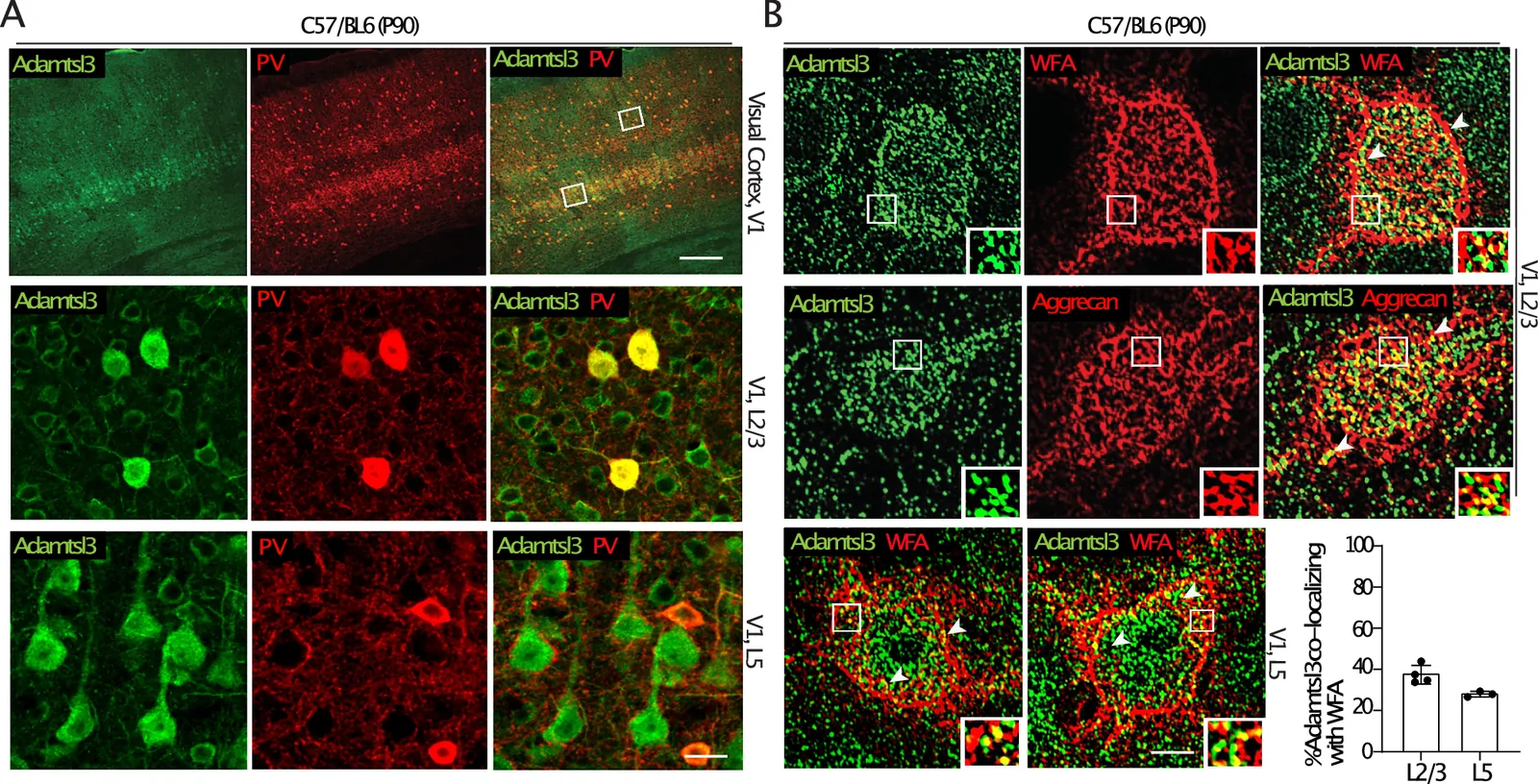
Adamtsl3 is associated with PNNs in V1. **A** Representative images showing Adamtsl3 (green) and parvalbumin (PV, red) expression in C57/BL6 mice V1 at P90. Scale bar 100 µm. Below: L2/3 and L5 zoom. Scale bar 20 µm. **B** Representative images showing Adamtsl3 (green), WFA or Aggrecan (red) at near-super resolution in L2/3 PV+ cells. Below: L5 shown. Arrows indicate puncta with spatial overlap (co-localization) of Adamtsl3 and PNN markers. Zoom in shown (white box). Scale bar 3 µm. Bottom right panel: quantification of Adamtsl3 puncta co-localzing with WFA marker (n = 3–4; 37 ± 3.9% in L2/3, 28 ± 1.1% in L5). Data are shown as mean ± SD.

To examine Adamtsl3 expression in V1, we used a previously validated home-made antibody [20] in wild-type (C57BL/6) mice. For all in vivo experiments, cohorts included both male and female mice, with sex balanced across experimental groups. At postnatal day 90 (P90), we observed Adamtsl3 expression in PV+ cells across all cortical layers of V1. In principal neurons, Adamtsl3 expression was lower and layer-specific, with relatively stronger labeling observed in layer 5 (L5) than in layer 2/3 (L2/3) neurons (Fig. 1A). To assess whether Adamtsl3 expression was associated with glial populations, we co-stained sections with the microglial marker Iba-1 and the astrocyte marker ALDH1L1 and observed no overt co-localization of Adamtsl3 immunoreactivity with either marker (Fig. S1). These findings are consistent with glial transcriptomic datasets, which report negligible *Adamtsl3* expression in microglia, and astrocytic *Adamtsl3* mRNA only emerging under reactive inflammatory conditions [22].

To assess Adamtsl3 localization to PNNs, we co-stained with Wisteria floribunda agglutinin (WFA), which binds N-acetylgalactosamine β1 residues in PNN glycoproteins, and aggrecan, a major structural component of PNNs [23, 24]. Near super-resolution imaging revealed Adamtsl3 puncta that co-localized with both PNN markers across cortical layers (Fig. 1B, two representative cells shown per layer). Quantitative analysis showed that 37 ± 3.9% of Adamtsl3 puncta co-localized with WFA in L2/3 and 28 ± 1.1% in L5 (Fig. 1B). These findings indicate that Adamtsl3 is associated with PNNs in adult V1.

**Fig. 2:**
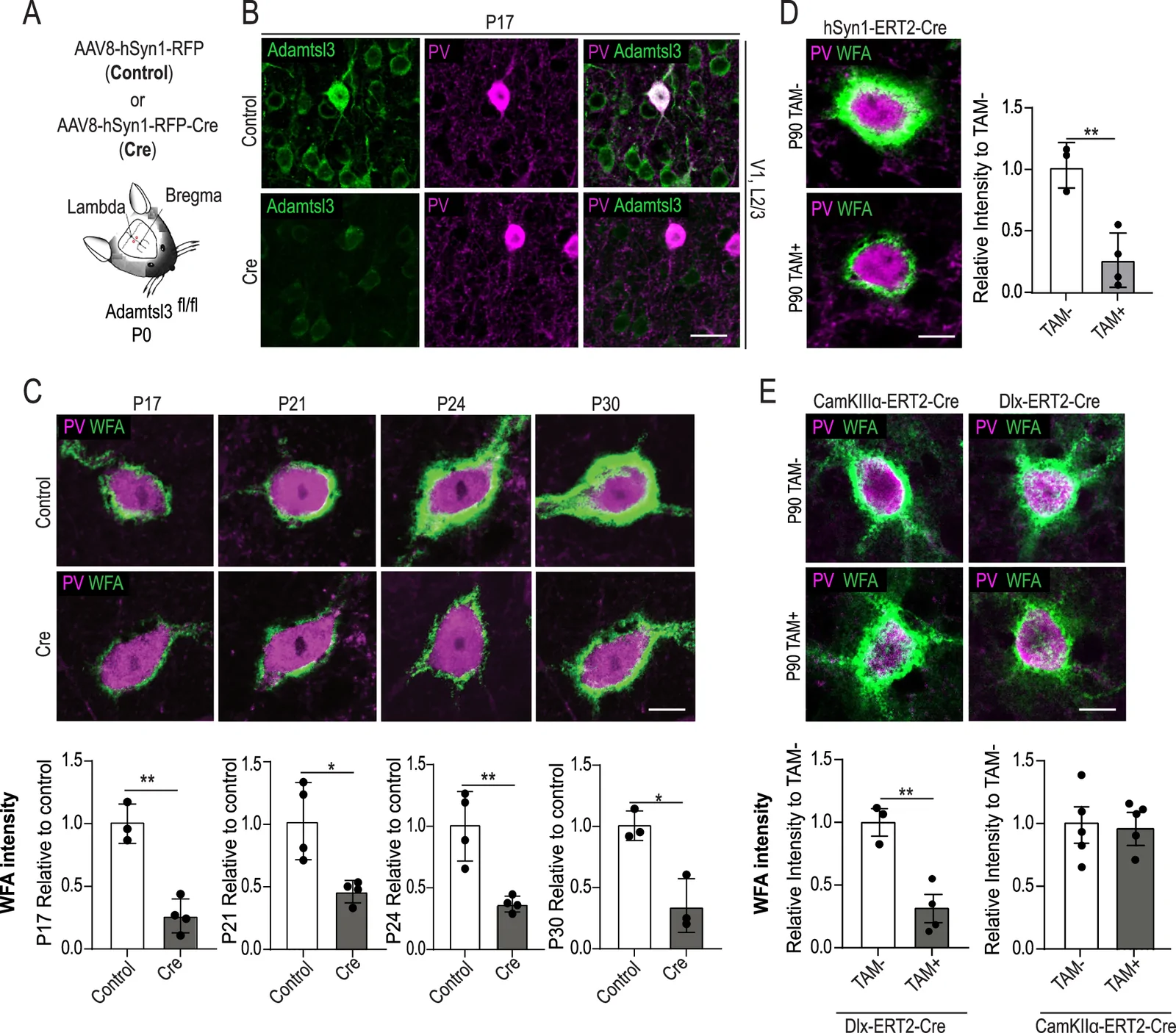
Adamtsl3 influences PNN maturation and adult maintenance in V1. **A** Illustration of P0 lateral ventricle injection. **B** Representative images showing reduction of Adamtsl3 (green) in cre expressing PV+ (magenta) cells at P17. Scale bar 20 µm. **C** Representative images showing reduction of WFA (green) around L2/3 PV+ cells (magenta) in AAV8-hSyn1-RFP-Cre injected animals at P17, P21, P24, and P30. Below: quantification relative to control (n = 3–4, 15 PV+ cells/ animal). **D** Representative images showing reduction of WFA (green) around L2/3 PV+ cells (magenta) in TAM + AAV8-hSyn1-ERT2-Cre injected animals. Right: quantification relative to TAM- controls (n = 3–4, 15 PV+ cells/ animal). **E** Representative images showing reduction of WFA (green) around L2/3 PV+ cells (magenta) in TAM + AAV8-hDlx-ERT2-Cre injected animals: Below: quantification relative to TAM- controls (n = 3–4, 15 PV+ cells/ animal). Scale bar 10 µm. Data is shown as mean ± SD. Student’s two-tailed t-test, * P < 0.05, ** P < 0.01.

PNN formation coincides with CPs during early postnatal development, with mature PNNs emerging around P30 as the CP begins to close by ~P60-90 in V1 [1, 21]. To investigate a role for Adamtsl3 in PNN maturation, we deleted *Adamtsl3* by injecting adeno-associated viruses (AAV) AAV8-hSyn1-RFP-Cre (Cre) or AAV8-hSyn1-RFP (control) into the lateral ventricles of *Adamtsl3*flox/flox pups at P0 (Fig. 2A). This approach enabled broad transduction across cortical layers and robust Cre expression by P17 (Fig. S2A), consistent with previous reports [25, 26]. We focused on L2/3, where PV+ cells form local microcircuits with principal cells [27]. Immunostaining confirmed a marked reduction in Adamtsl3 expression in Cre-injected animals at P17 (Fig. 2B), validating the efficiency of our gene-ablation strategy. To assess the impact on PNN maturation, we stained for WFA (Fig. 2C) and aggrecan (Fig. S2B) at key developmental stages of CP onset in V1 (P17, P21, P24, and P30). Signal intensities of both markers were significantly reduced surrounding L2/3 PV+ cells in Cre-injected mice compared to controls (Figs. 2C & S1B), indicating that Adamtsl3 contributes to PNN maturation in V1.

To assess the role of Adamtsl3 in maintaining PNN integrity in the adult brain, we utilized a tamoxifen-inducible Cre system (AAV8-hSyn1-ERT2-Cre) injected into the lateral ventricles of *Adamtsl3*^*flox/flox*^ pups at P0. Cre recombinase expression was induced after CP closure (P60) via intraperitoneal administration of tamoxifen (TAM+) for five consecutive days, as previously described [28] (Fig. S2C). Analysis at P90 revealed reduced signal intensities for WFA (Fig. 2D) and aggrecan (Fig. S2D) in TAM+ animals compared to vehicle-treated (TAM-) controls. These findings indicate that Adamtsl3 supports not only PNN maturation but also the maintenance of PNN integrity in adult V1.

To investigate the neuron subtype-specific roles of Adamtsl3 in V1, we selectively deleted *Adamtsl3* in either principal cells or GABAergic interneurons. P0 *Adamtsl3*^*flox/flox*^ pups were injected with AAV expressing tamoxifen-inducible Cre recombinase driven by a principal cell-specific promoter [29] (AAV2/6-CaMKIIαERT2-Cre) or an interneuron-specific promoter [30] (AAV8-hDlxERT2-Cre) [20]. At P60, tamoxifen was administered intraperitoneally for five consecutive days to induce cell-type-specific recombination. WFA staining revealed intact PNNs after *Adamtsl3* deletion in principal neurons (Fig. 2E). In contrast, reduced PNN intensity was observed following interneuron-specific deletion (Fig. 2E). These results demonstrate an interneuron-specific role for Adamtsl3 in the formation and maintenance of PNNs in V1.

### Early loss of *Adamtsl3* disrupts PNN composition, Otx2 uptake, and redox balance

**Fig. 3:**
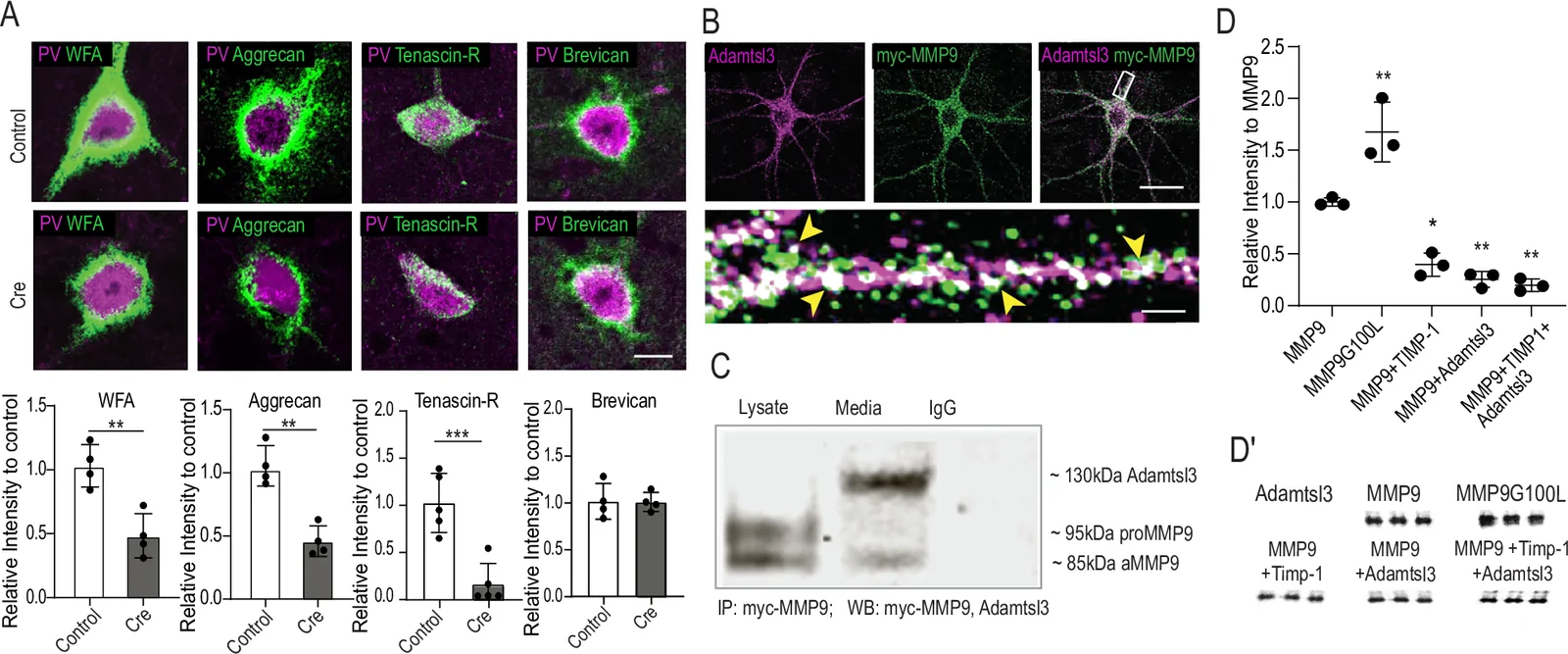
Adamtsl3 deletion compromises PNN integrity and influences MMP9 activity. **A** Representative images showing PV (magenta), WFA, aggrecan, tenascin-R, or brevican (green) expression in control and AAV8-hSyn1-RFP-Cre injected animals at P90. Below: quantification relative to control (n = 3–4, 15 PV+ cells/ animal). Scale bar 10 µm. Data is shown as mean ± SD. Student’s two-tailed t-test, ** P < 0.01, *** P < 0.001. **B** Primary rat neuron culture stained for myc-tag (myc-MMP9, green) and endogenous Adamtsl3 (magenta). Scale bar 20 µm. Below: zoom in. Scale bar 2 µm. Arrows indicate puncta with spatial overlap (co-localization) of Adamtsl3 and myc-MMP9. **C** Co-immunoprecipitation from HEK293 cells showing biochemical interaction between myc-MMP9 and eGFP-Adamtsl3 in serum-free media. **D** Gelatinase zymography of conditioned serum-free media from HEK293T cells expressing myc-MMP9 alone or co-expressing FLAG-TIMP-1 and/or eGFP-Adamtsl3. Gelatinolytic band intensity (zones of substrate proteolysis) reflecting MMP9 activity was normalized to the MMP9-alone condition. (n = 3; One-way ANOVA, Bonferroni multiple comparison test, F_(3,10)_ = 86.02, P < 0.0001). Data is shown as mean ± SD. (**D′**) Representative zymogram showing gelatinolytic activity bands under the indicated conditions (MMP9 alone, +TIMP-1, +Adamtsl3, +TIMP-1+Adamtsl3).

The molecular composition of PNNs is heterogeneous [31]. While WFA and aggrecan are widely used, to further assess the impact of *Adamtsl3* deletion on PNN structure, we surveyed several core PNN components in *Adamtsl3*^*flox/flox*^ mice injected at P0 with either AAV8-hSyn1-RFP-Cre (Cre) or control AAV8-hSyn1-RFP. Consistent with our prior observations in V1, Cre-injected mice continued to show significantly reduced WFA and aggrecan signals at P90 (Fig. 3A). Tenascin-R, another major PNN constituent, was also reduced at P90, whereas brevican levels were unaffected (Fig. 3A). These findings indicate that early postnatal loss of *Adamtsl3* alters PNN composition by affecting specific molecular components.

Next, we examined the expression of the transcription factor Otx2, whose PNN-mediated uptake promotes PV+ cell maturation and PNN maintenance through the CP [32, 33]. Immunolabelling revealed reduced Otx2 levels within L2/3 PV+ cells across CP timepoints and in P90 Cre-injected mice compared to controls (Fig. S3A). As postnatal Otx2 is synthesized in distant sources [32–34], we examined whether reduced Otx2 in cortical PV+ cells resulted from impaired PNN-mediated uptake or decreased retinal production. At P0, we injected *Adamtsl3*^*flox/flox*^ pups with either AAV8-hSyn1-RFP-Cre (Cre) or control AAV8-hSyn1-RFP in the lateral ventricles and collected cortical and retinal tissues at P30 for Otx2 quantification by Western blot. Cortical Otx2 levels remained unchanged, whereas retinal Otx2 levels were elevated in Cre-injected mice (Fig. S3B), perhaps reflecting a compensatory response to impaired PNN-mediated uptake. Together, these findings suggest that PNN disruption following early postnatal *Adamtsl3* deletion impairs Otx2 uptake into PV+ cells.

Enzymatic removal of PNNs has been shown to increase the vulnerability of adult PV+ cells to oxidative stress [35]. To determine whether *Adamtsl3* deletion affects redox balance, we labeled 8-oxo-7,8-dihydro-2’-deoxyguanosine (8-oxo-dG), a DNA adduct formed by guanine oxidation via hydroxyl radicals [36, 37]. While 8-oxo-dG levels in L2/3 PV+ cells were unchanged at P30, a significant increase was observed by P90 in Cre-injected mice compared to controls (Fig. S3C), pointing to a progressive accumulation of oxidative stress in adult PV+ cells. Together, these findings demonstrate that early postnatal deletion of *Adamtsl3* disrupts PNN composition and integrity, resulting in impaired Otx2 uptake and increased oxidative stress in adult PV+ cells.

### Adamtsl3 regulates MMP9 activity through two distinct functional domains

To investigate the mechanism underlying PNN reduction following *Adamtsl3* deletion, we examined MMP9, a known regulator of PNN integrity [7, 11, 19]. In rat primary neurons, endogenous Adamtsl3 and recombinant MMP9 (pCMV-myc-MMP9) at 7 days in vitro (DIV7) revealed protein co-localization at DIV14 (Fig. 3B). To confirm biochemical interaction, we performed co-immunoprecipitation in HEK-293T cells co-transfected with plasmids encoding GFP-Adamtsl3 and myc-tagged MMP9. MMP9 is synthesized intracellularly as an inactive proenzyme (~95 kDa) that is secreted and activated by cleavage of its pro-domain to generate the mature form (~85 kDa) [38]. While no interaction was detected in whole-cell lysates, we observed an interaction between Adamtsl3 and the active form of MMP9 in serum-free media (Fig. 3C), indicating that Adamtsl3 preferentially binds to the secreted, enzymatically active form of MMP9.

Next, we tested whether Adamtsl3 modulates MMP9 enzymatic activity using gelatin zymography. Serum-free media were collected from HEK-293T cells transfected with plasmids encoding pCMV-myc-MMP9, pBP84-eGFP-Adamtsl3, or pCMV-FLAG-TIMP1 (tissue inhibitor of metalloproteinase 1), a known endogenous inhibitor of MMP9. Cells transfected with an auto-activating MMP9 mutant MMP9G100L (pCMV-myc-MMP9G100L) served as a positive control [39]. Compared to MMP9 alone, activity was significantly reduced in the presence of either TIMP-1 or Adamtsl3 (Fig. 3D, D′), indicating that Adamtsl3 reduces MMP9 activity.

To identify domains within Adamtsl3 required for inhibiting MMP9, we generated five *Adamtsl3* deletion constructs (M#1–5) (Fig. S4A). In gelatin zymography assays, constructs M#2 (lacking the cysteine-rich spacer region) and M#5 (lacking thrombospondin repeat (TSR) domains 10–12) failed to reduce MMP9 activity, implicating these regions in Adamtsl3-mediated inhibition of MMP9 (Fig. S4B). Together, these results demonstrate that Adamtsl3 interacts with the active form of MMP9 in the extracellular space and modulates its enzymatic activity in vitro via distinct domains.

### Adamtsl3 maintains PNN integrity via MMP9 activity

**Fig. 4:**
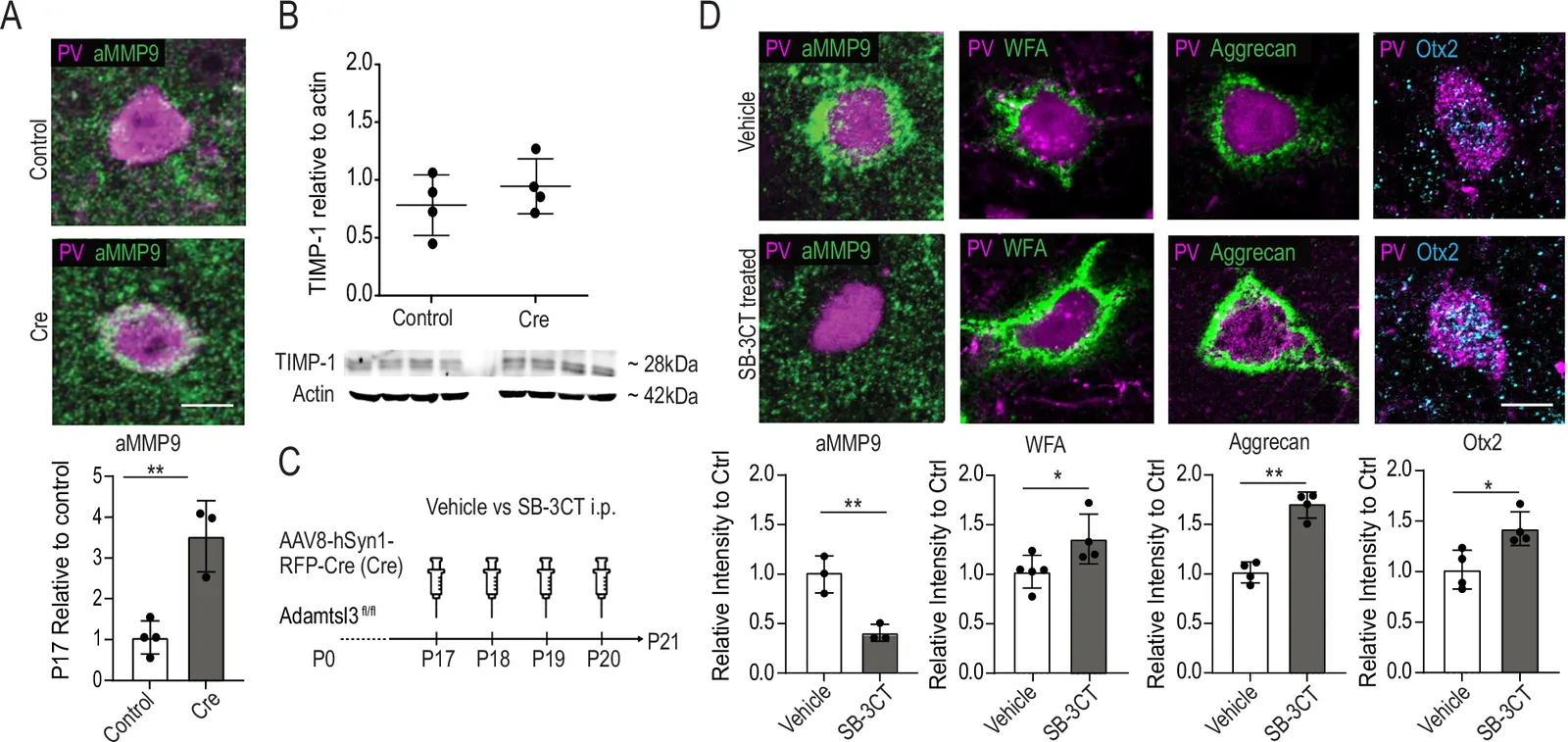
Adamtsl3 regulates MMP9 activity independent of TIMP-1 in vivo. **A** Representative images showing an increase in active MMP9 (green) around L2/3 PV+ cells (magenta) in AAV8-hSyn1-RFP-Cre injected animals at P17. Scale bar 10 µm. Below: quantification relative to control (n = 3–4, 15 PV+ cells/ animal). **B** Western blot for TIMP-1 in P30 cortical lysates (n = 4). **C** Timeline of SB-3CT experiment. **D** Representative images showing PV (magenta), active MMP9, WFA, aggrecan (green), and Otx2 (blue) expression after 4 days of vehicle or SB-3CT injection at P21. Below: quantification relative to vehicle control (n = 3–4, 15 PV+ cells/ animal). Scale bar 10 µm. Data is shown as mean ± SD. Student’s two-tailed t-test, * P < 0.05, ** P < 0.01.

To assess the role of Adamtsl3 in regulating MMP9 activity in vivo, we used immunostaining to detect the active (processed) form of MMP9 in *Adamtsl3*^*flox/flox*^ mice injected at P0 with AAV8-hSyn1-RFP-Cre (Cre) or AAV8-hSyn1-RFP (control). In Cre-injected mice, active MMP9 levels were significantly elevated around L2/3 PV+ cells at P17 compared to controls (Fig. 4A), and this increase persisted at P24, P30, and P90 (Fig. S4C). Because MMP9 activity is typically regulated by its endogenous inhibitor TIMP-1 [40], we examined whether changes in TIMP-1 expression could account for this increase. Western blot analysis of cortical tissue at P30 showed no difference in TIMP-1 levels between Cre-injected and control animals (Fig. 4B). These results indicate that *Adamtsl3* deletion increases MMP9 activity in vivo through a mechanism independent of TIMP-1 levels.

To test whether elevated MMP9 activity drives PNN disruption in *Adamtsl3*-deficient mice, we treated animals with the synthetic gelatinase inhibitor SB-3CT [41, 42]. *Adamtsl3*^*flox/flox*^ mice injected with AAV8-hSyn1-RFP-Cre at P0 were treated with SB-3CT (i.p.) for four consecutive days before the CP (P17–P20), and PNN integrity and Otx2 uptake were assessed at P21 (Fig. 4C). Active MMP9 levels were significantly reduced in Cre-injected mice treated with SB-3CT compared with vehicle-treated controls, while WFA, aggrecan, and Otx2 markers were elevated (Fig. 4D). These results demonstrate that Adamtsl3 functions as an endogenous brake on MMP9 in vivo, and that PNN deficits caused by *Adamtsl3* deletion can be rescued by pharmacological blockade of MMP9.

### Adamtsl3 functions autonomously within PV+ cells

**Fig. 5:**
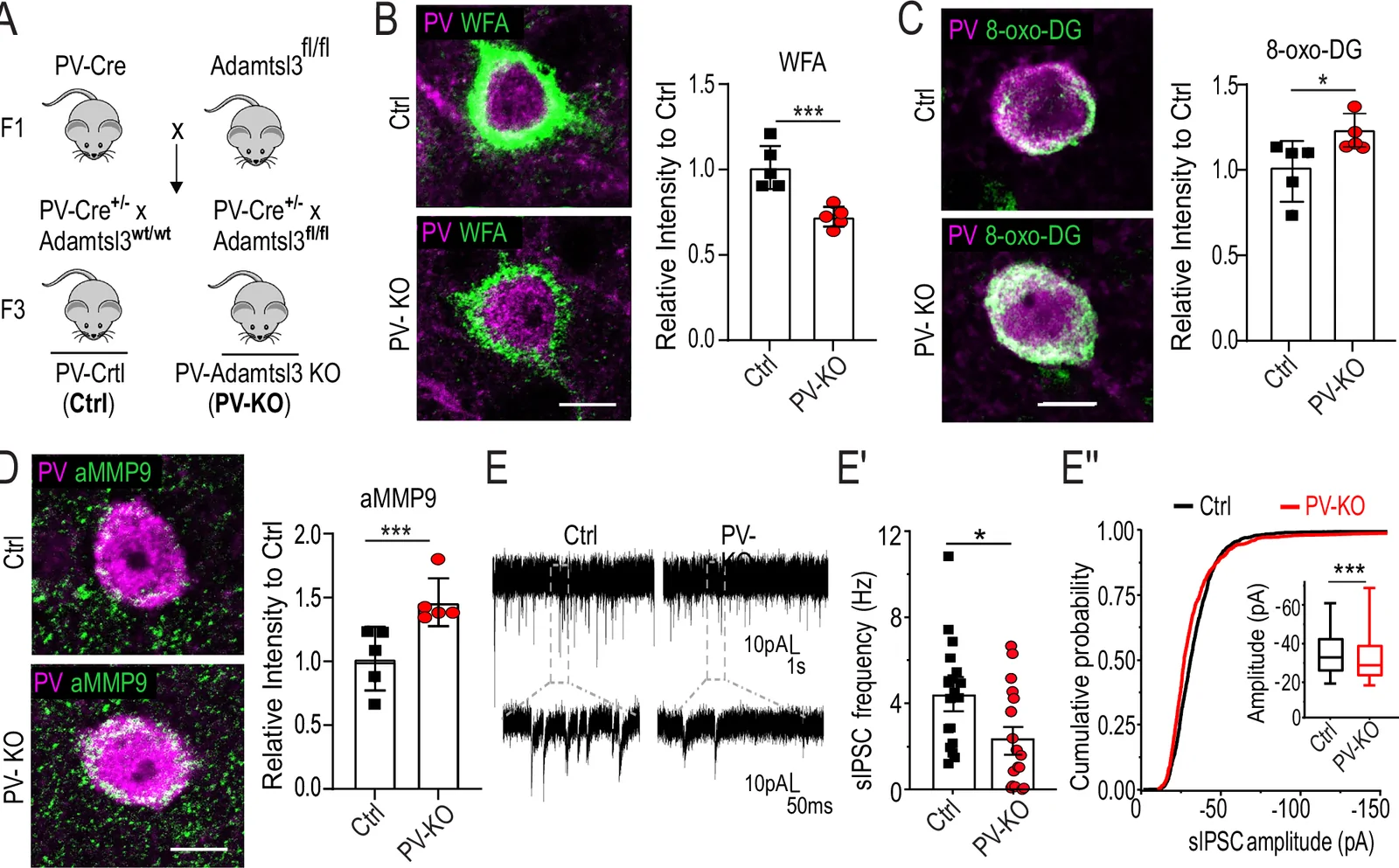
PV cell-specific deletion of Adamtsl3 disrupts PNNs and GABAergic neurotransmission. **A** Breeding strategy for PV-Cre control (Ctrl) and PV-Adamtsl3 KO (PV-KO). **B**-**D** Representative images showing **B** WFA (green), **C** 8-oxo-dG (green), and **D** active MMP9 (green) expression around L2/3 PV+ cells (magenta) in PV-KO at P120. Right: quantification relative to controls (n = 5, 15 PV+ cells/ animal). Scale bar 10 μm. Data is shown as mean ± SD. Student’s two-tailed t-test, * P < 0.05, *** P < 0.001. **E** Representative spontaneous inhibitory postsynaptic currents (sIPSCs) from L2/3 PV-KO pyramidal neurons. **E′** sIPSC frequency (p = 0.0161), n = 11–18 cells. Data are shown as mean ± SEM. Student’s two-tailed t-test, *P < 0.05. **E″** Cumulative probability distributions of sIPSC amplitudes. Insert: box plot showing median, 5–95%, and interquartile ranges of sIPSC amplitudes (p = 3.922e-10, Mann-Whitney test), 943-1789 events.

To confirm whether Adamtsl3 functions autonomously within PV+ cells to regulate PNNs, we crossed *Adamtsl3*^*flox/flox*^ mice with a PV-Cre driver line to generate PV-specific *Adamtsl3* knockout (PV-KO) mice (Fig. 5A). At P120, we validated PV+ cell-specific deletion of *Adamtsl3* using in situ hybridization (Fig. S5A) and by immunolabelling (Fig. S5B), which showed a near-complete loss of Adamtsl3 signal in PV+ cells. To confirm PV-specific *Adamtsl3* deletion, we stained V1 for Adamtsl3 in L5 pyramidal neurons, confirming preserved expression in non-PV+ neurons (Fig. S5B′). PV-Cre control (Ctrl) and PV-KO mice were viable, fertile, and produced litters in Mendelian ratios with no abnormalities. Quantification of PV+ cell density in V1 revealed no differences between genotypes, indicating that *Adamtsl3* deletion does not affect interneuron migration or survival to P120 (Fig. S5C). Analysis of PNN-associated markers showed reduced WFA staining in L2/3 PV+ cells (Fig. 5B) and significantly elevated levels of the oxidative stress marker 8-oxo-dG (Fig. 5C) and active MMP9 (Fig. 5D) in PV-KO mice compared to controls. These findings establish that Adamtsl3 functions autonomously within PV+ cells to regulate PNN integrity and MMP9 activity.

### PV cell-specific deletion of *Adamtsl3* disrupts GABAergic neurotransmission

PNN disruption has been linked to alterations in the electrophysiological properties of PV+ cells and nearby pyramidal neurons [43–45]. To assess the functional consequences of PV+ cell-specific *Adamtsl3* loss in PV-KO mice, we first examined PV+ cell synapse density by labeling for Synaptotagmin 2 (Syt2), a marker of PV+ presynaptic terminals [46]. In naive mice, PNN maturation correlates with an increase in PV+ cell synapses, both onto other PV+ cells and onto pyramidal neurons [47]. In PV-KO mice, Syt2+ terminals were reduced on PV+ cell somata (Fig. S5D) and more broadly in L2/3 (Fig. S5D′). These findings suggest that PNN deficits caused by *Adamtsl3* deletion reduce both PV+ to PV+ cell connectivity and inhibitory input from PV+ cells onto L2/3 pyramidal neurons.

To corroborate these findings, we performed whole-cell recordings of spontaneous inhibitory postsynaptic currents (sIPSC) from L2/3 pyramidal neurons in adult mice (P ~ 120–210). In PV-KO mice, the frequency and amplitude of sIPSC were significantly reduced compared to controls (Fig. 5E-E″), indicating reduced inhibition. Although PNN disruption may variably impact PV-circuit output across contexts, reduced inhibitory drive has been observed in other paradigms [48, 49], consistent with our findings in V1.

### Adult PV cell-specific deletion of *Adamtsl3* reinstates juvenile-like plasticity in V1

**Fig. 6:**
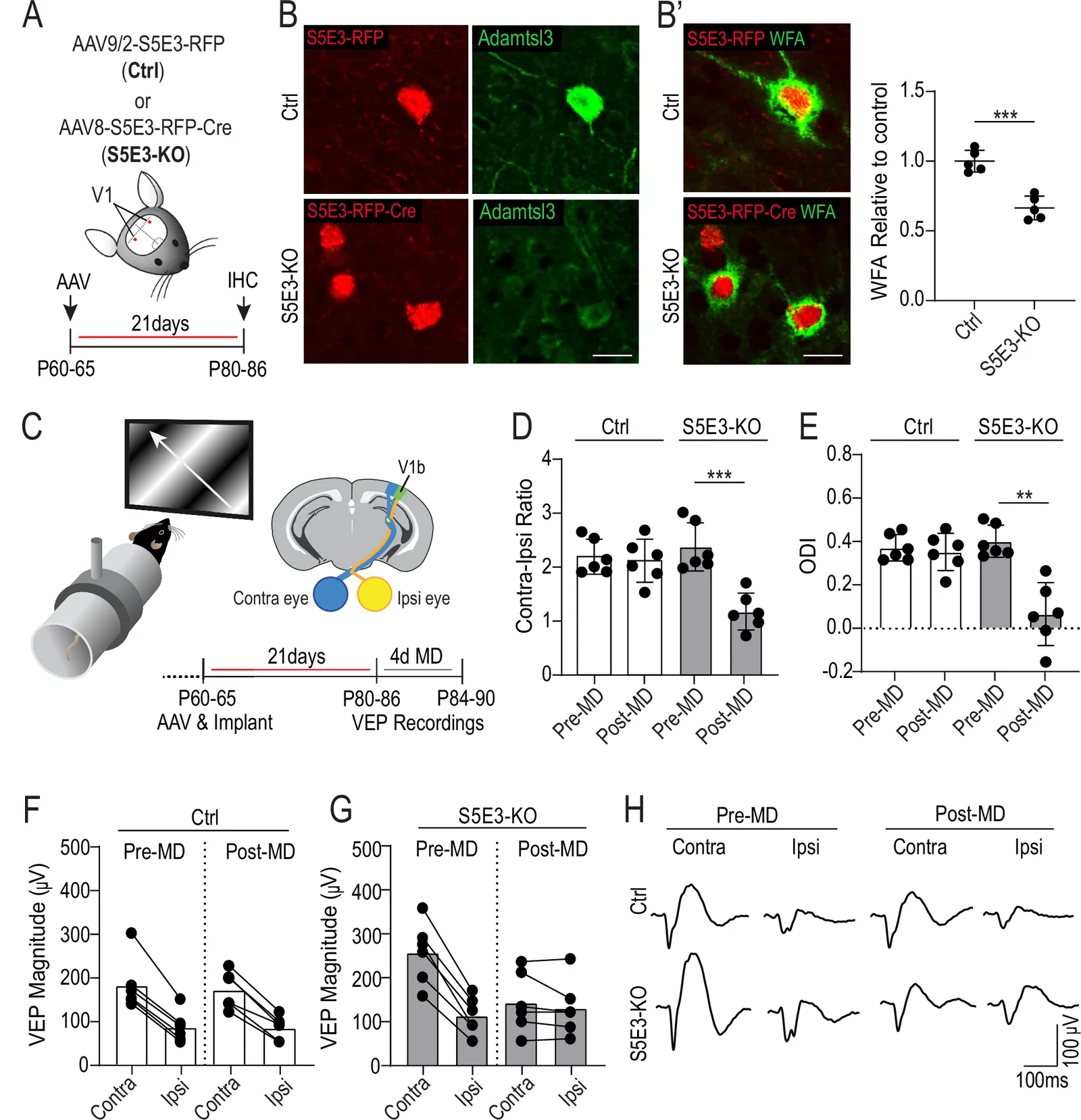
Adult PV cell–specific deletion of Adamtsl3 reinstates juvenile-like plasticity in V1. **A** Illustration and timeline of AAV9/2-S5E3-RFP (Ctrl) or AAV9/2-S5E3-RFP-Cre (S5E3-KO) injection into V1. **B** Representative images showing S5E3-RFP (red) and Adamtsl3 (green) expression in V1 of control and S5E3-KO animals 21 days post injection. Scale bar 20 µm. **B**′ Representative images showing S5E3-RFP (red) and WFA (green) labeling in V1 of control and S5E3-KO animals. Right: quantification of WFA intensity relative to control (n = 5, 15 S5E3 + /PV+ cells/ animal). **C** Experimental timeline to assess ocular dominance (OD) plasticity in adult mice. **D** Contra-/ipsilateral eye ratios before (Pre-MD) and after (Post-MD) MD in control and S5E3-KO animals. **E** Ocular dominance index (ODI) before and after MD in control and S5E3-KO animals. **F**–**G** Mean monocular VEP magnitudes (µV) evoked by contra- and ipsilateral eye stimulation in control **F** and S5E3-KO mice **G** animals before and after MD (n = 6). **H** Averaged VEP traces from control and S5E3-KO animals. Data is shown as mean ± SD. Student’s two-tailed t-test, ** P < 0.01, *** P < 0.001.

Given the well-established role of PNNs in restricting experience-dependent plasticity [1, 10], we investigated whether *Adamtsl3* deletion alone could reinstate juvenile-like plasticity in adult V1. To selectively affect PV+ cells, we stereotactically injected AAV9/2-S5E3-RFP-Cre (S5E3-KO) or AAV9/2-S5E3-RFP (Ctrl) into V1 of P60-65 *Adamtsl3*^*flox/flox*^ mice (Fig. 6A) and allowed 20–21 days for robust viral expression (Fig. S6A). Consistent with previous reports [50], the S5E3 promoter effectively targeted PV+ cells, with over 80% of RFP⁺ cells co-expressing PV (Fig. S6B). Immunostaining confirmed Adamtsl3 reduction (Fig. 6B) and a significant loss of WFA labeling around L2/3 PV⁺/Cre⁺ cells (Fig. 6B′) spanning V1 (Fig. S6C), validating the PV-target specificity and effective dismantling of PNNs by our gene ablation strategy.

We next investigated whether *Adamtsl3* deletion altered baseline visual responses in adult V1. We injected V1 of P60–65 *Adamtsl3*^*flox/flox*^ mice with either AAV9/2-S5E3-RFP-Cre (S5E3-KO) or AAV9/2-S5E3-RFP (Ctrl) virus. Visual evoked potentials (VEPs) recorded from layer 4 of binocular V1 at 20–21 days post-injection revealed significantly elevated absolute VEP amplitudes in S5E3-KO mice (Figs. S6D, 3E, E′). This finding is consistent with reduced GABAergic activity (Fig. 5E), as evidenced by cortical disinhibition and elevated baseline VEP responses [43, 51].

To directly assess whether PV-specific *Adamtsl3* deletion reinstates juvenile-like plasticity, we performed a standard 4-day monocular deprivation (MD) protocol to measure ocular dominance (OD) in adult mice [52–54]. In S5E3-KO or Ctrl mice prepared as above, VEPs were recorded 20-21 days post-injection both before and 4 days after MD (Fig. 6C). In normally reared adults [52], responses driven by the contralateral eye are typically 2–3 times larger than those from the ipsilateral eye, resulting in a contralateral-to-ipsilateral (C/I) VEP ratio between 2 and 3 and an ocular dominance Index (ODI) between 0.3 and 0.5.

In Ctrl mice, there was no effect of 4-day MD on either the C/I ratio (Fig. 6D) and ODI (Fig. 6E) or the absolute amplitude of VEPs (Fig. 6F, H) evoked by the two eyes, consistent with a lack of OD plasticity in adult V1. In contrast, S5E3-KO mice exhibited a significant OD shift after 4 days of MD, with the C/I ratio shifting towards the non-deprived (ipsilateral) eye (Fig. 6D, E). This shift was entirely driven by a reduction in the deprived contralateral eye response (Fig. 6G, H), closely resembling the plasticity observed in juvenile mice around P30 [52]. These findings demonstrate that selective deletion of *Adamtsl3* from adult PV+ cells is sufficient to restore juvenile-like plasticity in adult mouse V1, as expected when PNNs are dismantled [1, 10, 51].

## Discussion

The formation of PNNs during CPs is crucial for limiting experience-dependent plasticity, stabilizing inhibitory circuits, and maintaining the balance between excitatory and inhibitory neurotransmission. In this study, we identify Adamtsl3 as a glycoprotein associated with PNNs that is essential for both their developmental maturation and adult maintenance in V1. Notably, large-scale genome-wide association studies have robustly implicated ADAMTSL3 as a schizophrenia risk gene [55, 56], providing a strong rationale to investigate how ADAMTSL3 regulates extracellular matrix organization and inhibitory circuit stability in the cortex. By using conditional deletion of *Adamtsl3* at specific developmental stages and in specific neuronal subtypes, we demonstrate that Adamtsl3 plays a vital role in both PNN maturation and maintenance. Furthermore, Adamtsl3 functions as an extracellular inhibitor of MMP9, which helps to constrain experience-dependent plasticity in the adult V1.

### Adamtsl3 and MMP9 Regulation

MMPs, particularly MMP9, play a crucial role in the remodelling of the ECM and PNNs in the postnatal brain. While MMP9 activity is typically regulated by TIMPs, such as TIMP-1, which binds both pro- and active MMP9 [17, 18], additional modulators, including thrombospondins, have been implicated in fine-tuning MMP9 activity [19]. In this study, we have identified Adamtsl3 as an extracellular inhibitor of MMP9. Unlike TIMP-1, Adamtsl3 selectively interacts with the active, secreted form of MMP9 in the extracellular space, thereby reducing its enzymatic activity independently of TIMP-1. This inhibitory function is mediated by two critical Adamtsl3 domains: the cysteine-rich spacer region and C-terminal TSRs 10–12. Both regions are highly conserved within the ADAMTS superfamily and have been implicated in MMP regulation by related family members, such as ADAMTS-1 [57], ADAMTS-4 [58], and ADAMTS-13 [59]. Notably, TSR domains are known to inhibit MMPs [60].

These findings provide a mechanistic framework for interpreting human genetic associations at ADAMTSL3, linking schizophrenia-associated variation to a defined molecular mechanism that impacts PNN integrity. Notably, the schizophrenia-associated variant rs950169 (T1663I) is predicted to generate a truncated Adamtsl3 protein lacking C-terminal thrombospondin type 1 repeats (TSR10–12) [15]. Within the broad context of altered gene expression observed in cortical and hippocampal tissue from individuals with schizophrenia [61], and GWAS evidence implicating ADAMTSL3 among ECM‑related risk genes [62], this variant is likely to have functional consequences in brain regions critical for cognition and sensory processing. Loss of the C-terminal TSR domains would be expected to impair Adamtsl3-mediated inhibition of MMP9, thereby promoting excessive extracellular matrix proteolysis. Such dysregulation provides a plausible mechanistic link between ADAMTSL3 genetic risk, PNN destabilization, and PV+ interneuron dysfunction in schizophrenia [35]. Consistent with this model, overactivation of the MMP9–RAGE signaling pathway has been shown to drive oxidative stress and inhibitory–excitatory imbalance in both animal models and schizophrenia patients, and pharmacological inhibition of MMP9 has been reported to restore PNN integrity and promote PV+ interneuron maturation [63].

Although our data suggest a specific role for Adamtsl3 in regulating MMP9, its inhibitory profile may extend to other MMPs, such as the closely related MMP2. Nevertheless, MMP9 was the only metalloproteinase found to show mRNA changes in response to monocular deprivation [64], highlighting its role as the key ECM-remodelling enzyme linked to experience-dependent plasticity in V1. While our study focuses on PNN-associated MMP9, both Adamtsl3 and MMP9 are expressed in other neural compartments [20, 65]. Therefore, Adamtsl3 may also regulate MMP activity beyond PNNs, potentially influencing extracellular remodelling in the diffuse ECM as well as synaptic remodelling and dendritic structure.

### Cell-Autonomous Roles of Adamtsl3 in PV+ Cell PNN Maintenance

While PV+ cells play a crucial role in maintaining their own PNN integrity through cell-autonomous mechanisms [51], external factors also contribute to PNN stability [32, 34, 66]. Given that Adamtsl3 is expressed in both principal cells and PV+ interneurons in V1, we employed a PV-specific *Adamtsl3* knockout (PV-KO) strategy to investigate its cell-autonomous function. Our findings revealed that Adamtsl3 within PV+ cells is essential for preserving PNN integrity and GABAergic neurotransmission onto L2/3 pyramidal neurons. We focused on layer 2/3 pyramidal neurons because somatic GABAergic responses in these cells provide a sensitive readout of the plastic state of primary visual cortex [67], a process strongly shaped by PV+ interneuron function [4]. Consistent with this, prior work has indicated that changes in somatic GABA_A_ receptor–mediated synaptic efficacy at PV-pyramidal synapses are critical for regulating experience-dependent plasticity [68]. Thus, considering our PV- specific knockout approach and the established PV contribution to L2/3 somatic inhibition [67, 68], the observed sIPSC changes most likely reflect altered PV+ interneuron function.

In a complementary approach, conditional deletion of *Adamtsl3* in adult PV+ cells (S5E3-KO) also led to a significant reduction in PNN integrity and a substantial increase in baseline visual evoked potential (VEP) amplitudes, consistent with reduced inhibitory control. Prior work indicates that the functional consequences of PNN disruption for PV-circuit output are heterogeneous and context dependent. Prolonged PNN degradation has been associated with reduced PV excitability and weakened inhibitory drive, as well as a shift of V1 networks toward a more disinhibited, juvenile‑like state [43, 48, 69–71]. In contrast, others observed no changes in PV-pyramidal cell transmission but instead identified reductions in inhibitory input onto PV cells, FosB expression in PV interneurons, and gamma oscillatory activity [72, 73]. In our study, PV-specific *Adamtsl3* deletion was accompanied by reduced inhibitory drive onto L2/3 pyramidal neurons and increased VEP amplitudes, supporting the idea that Adamtsl3 functions within PV+ cells to regulate MMP9 activity and PNN integrity, with downstream consequences for inhibitory circuit function. We further identified reduced Otx2 uptake in PV+ cells during development and elevated levels of oxidative stress markers in adulthood, which could be downstream consequences of reduced PNN integrity. These findings align with prior evidence implicating PNNs in supporting PV+ cell function by facilitating Otx2 uptake and reducing oxidative stress [32, 34, 35]. Pharmacological inhibition of MMP9 using SB-3CT partially restored PNN integrity and increased internalized Otx2 levels, emphasizing the therapeutic potential of targeting MMP9 in conditions where PNN disruption is implicated [9, 63].

We have previously shown that Adamtsl3 localizes to inhibitory presynaptic terminals in the hippocampus, where it regulates GABAergic neurotransmission via deleted in colorectal cancer (DCC) signalling [20]. Thus, we cannot exclude the possibility that the reduced inhibition observed here partly reflects a loss of presynaptic Adamtsl3 function, independent of PNN integrity. However, several aspects of the current data identifies a role for Adamtsl3 at PNNs: (1) PNN deficits caused by *Adamtsl3* deletion can be rescued by pharmacological inhibition of MMP9, indicating that Adamtsl3 acts upstream of MMP9 in the PNN maintenance pathway; (2) the electrophysiological phenotype of both PV-KO and S5E3-KO mice mirrors that produced by enzymatic PNN removal [43–45, 51]; and (3) the PV+-specific deletion spares other inhibitory populations in V1, suggesting a localized, PNN-dependent mechanism.

We further propose that Adamtsl3 isoforms differentially contribute to the results presented here and in Cramer et al. [20]. In mice, three splice variants have been described, two of which contain a truncated C-terminal PLAC domain [15], a structural motif implicated in extracellular matrix interactions [74, 75]. Therefore, distinct isoforms may account for Adamtsl3’s compartment-specific functions at PNNs versus presynaptic sites. However, a key limitation of the present study is that our current antibody is directed against an N-terminal epitope and therefore does not distinguish between isoforms. Consequently, all immunolabeling and biochemical signals reported here likely reflect a composite of multiple Adamtsl3 isoforms. As a result, the relative contributions of individual isoforms to the PNN phenotype versus synaptic effects remain unresolved in the current study. Understanding how alternative splicing influences Adamtsl3 localization, its interaction with DCC, and its regulation of MMP9 will be crucial for disentangling its distinct roles at synapses and at the PNN. Notably, the schizophrenia-associated *Adamtsl3* variant is predicted to generate a truncated C-terminal [15], likely compromising its localization and function at PNNs. Such disruptions could contribute to PNN deficits and the associated PV+ interneuron dysfunction observed in schizophrenia.

### Adamtsl3 and experience-dependent plasticity

Given the well-established role of PNNs in restricting experience-dependent plasticity [10, 51], we investigated whether *Adamtsl3* deletion alone could reinstate juvenile-like plasticity in adult V1. We found that PV-specific deletion of *Adamtsl3* reduced PNNs and restored juvenile-like OD plasticity in adulthood. This supports the critical role of PNNs as gatekeepers of cortical plasticity. This finding aligns with previous studies showing that enzymatic degradation of PNNs using chondroitinase ABC shifts OD in adult V1 [43, 76]. MMP9 has also been directly implicated in OD plasticity: MMP9 knockout mice exhibit impaired plastic responses to MD during the CP [77], and MMP9 activity is required for reactivation of plasticity following antidepressant treatment in adults [78]. Together with our observation that Adamtsl3 inhibits MMP9 activity, these findings strongly suggest that elevated MMP9 activity following Adamtsl3 deletion provides the mechanistic basis for the reinstatement of OD plasticity in adulthood. While we cannot exclude the possibility that reduced inhibition due to loss of presynaptic Adamtsl3 function in PV+ cells contributes to the observed OD plasticity, the close phenotypic overlap between *Adamtsl3* deletion and PNN-disrupting manipulations supports a PNN-mediated mechanism. The induction of robust OD plasticity after *Adamtsl3* deletion, driven by reduced responses to the deprived eye, as observed in juveniles [52], underscores the essential role of the Adamtsl3–MMP9 brake in maintaining the PNN barrier to experience-dependent circuit remodeling after the CP. Dysregulation of this axis may therefore contribute to pathological plasticity or impaired critical-period closure in neurodevelopmental disorders such as schizophrenia [35, 62].

## Materials and methods

**Key Resources Table**

**Table:** 

Reagent or Resource	Source	Identifier
**Antibodies**		
Actin	Millipore	Cat no. MAB1501
Adamtsl3	Home-made	Cramer et al. [20]
Aggrecan	Merck	Cat no. MAB1581
Cre	Biolegend	Cat no. PRB-106P
FLAG (6F7)	Sigma	Cat no. SAB4200071
IBA-1	WAKO	Cat no. 019-19741
Myc (9E10)	Roche/Sigma	Cat no. 11 667 149 001
MMP9 (active)	Merck	Cat no. MABT171
OTX2	Abcam	Cat no. ab114138
Parvalbumin	Immunostar	Cat no. 24428
Parvalbumin	Swant	Cat no. PV25
Parvalbumin	Sigma	Cat no. P3088
TIMP-1	Millipore	Cat no. MAB9801
Tenascin R	Thermo Fisher	Cat no. PA5-47546
Synaptotagmin 2	Synaptic Systems	Cat no. 105 123
WFA, biotinylated	Adipogen	Cat no. VC-B-1355-M002
8-oxo-dG	R&D	Cat no. 4354-MC-050
Iba-1	Cell Signalling	Cat no. 58970
ALDH1L1	NeuroMap	Cat no. 75-140-020
**Virus strains**		
AAV8- hSyn1-RFP-Cre	Viral Vector Facility, UZH/ETH	Cat no. v230
AAV8- hSyn1-RFP	Viral Vector Facility, UZH/ETH	Cat no. v133
AAV8- hSyn1-ERT2-Cre	Viral Vector Facility, UZH/ETH	Cat no. v325
AAV8- hDlx-ERT2-Cre	Viral Vector Facility, UZH/ETH	Cat no. v301
AAV2/6- CAMKIIα-ERT2-Cre	Vector biolabs	Cat no. 2014-1208
AAV9/2-S5E3-RFP-Cre	Viral Vector Facility, UZH/ETH	v1087-9
or AAV9/2-S5E3-RFP	Viral Vector Facility, UZH/ETH	v1082-9
**Chemicals, Peptides, and Recombinant Proteins**		
Tamoxifen	Sigma	Cat no. T5648
Complete Mini Protease Inhibitor	Roche	Cat no. 1183617001
Phosphatase inhibitor cocktail 2, 3	Sigma	Cat no. P0044, p5926
Pierce Protein A/G Ultralink Resin	Thermo Fisher	Cat no. 53132
Western Blocking Reagent	Roche	Cat no. 11921673001
SuperSignal™ West Pico PLUS Chemiluminescent Substrate	Thermo Fisher	Cat no. 34577
Gelatin	Thermo Fisher	Cat no. AC612255000
Neurobasal Plus media	Thermo Fisher	Cat no. 836
OCT compound	Sakura	Cat no. 4583
SB-3CT	APExBIO	Cat no. B4792
Kynurenic acid	Sigma	Cat no. K3375
**Critical Commercial Assays**		
RNAscope Fluorescent Multiplex Detection Kit	ACDbio	Cat no. 320851
**Software and Algorithms**		
Synaptic cluster analysis Python-script ImageJ	Cramer et al. [25]	https://github.com/dcolam/Cluster-Analysis-Plugin
Clampfit ver 10.7	Molecular devices	https://www.moleculardevices.com/
WinEDR ver 4.0.2	John Dempster	https://spider.science.strath.ac.uk/sipbs/software_ses.htm
WinWCP ver 5.7.0	John Dempster	https://spider.science.strath.ac.uk/sipbs/software_ses.htm
Originpro 2021b	OriginLab Corporation	https://www.originlab.com/2021
Graphpad Instat ver 3.06	Graphpad software	https://www.graphpad.com/scientific-software/instat/
**Experimental Models: cell lines**		
Human: HEK293T	ATCC	Cat no. CRL-3216
**Experimental Models: organisms/strains**		
Mouse: Adamtsl3^flox/flox^	Cyagen, USA	C57BL/6 background Cramer et al. [20]
Mouse: PV-Cre	Jackson Laboratory	Cat no. 008069
**Recombinant DNA**		
Plasmid: C-terminal eGFP-tagged Adamtsl3	Berangere Pinan-Lucarre	Cramer et al. [20]
Plasmid: N-terminal FLAG-tagged TIMP-1	Sino Biological Inc.	Cat no. HG10934-NF
Plasmid: C-terminal myc-tagged MMP9	Origene	Cat no. RC202872
Plasmid: C-terminal myc-tagged MMP9 (G100L)	Home-made	custom made
Plasmid: C-terminal eGFP-tagged Adamtsl3	Vector Builder	custom made
Plasmid: C-terminal eGFP-tagged Adamtsl3 (Δ PLAC)	Vector Builder	custom made
Plasmid: C-terminal eGFP-tagged Adamtsl3 (Δ TSR1-Cysteine)	Vector Builder	custom made
Plasmid: C-terminal eGFP-tagged Adamtsl3 (Δ TSR2-9)	Vector Builder	custom made
Plasmid: C-terminal eGFP-tagged Adamtsl3 (Δ Ig-like domain)	Vector Builder	custom made
Plasmid: C-terminal eGFP-tagged Adamtsl3 Δ TSR10-12	Vector Builder	custom made
RNA probe: Mm-Adamtsl3	ACDbio	Cat no. 474891
RNA probe: Mm-Parvalbumin	ACDbio	Cat no. 421931

### Animals and ethics statement

All procedures fulfilled the ARRIVE guidelines on experimental design, animal allocation to different experimental groups, blinding of samples to data analysis and reporting animal experiments. All procedures on animals were conducted in accordance with the European Directive 86/609 (EEC Council for Animal Protection in Experimental Research and Other Scientific Utilization), approved by the cantonal veterinary office of Zurich, the institutional animal care and use committees of Harvard University, Boston Children’s Hospital and MIT and followed the guidelines of the National Institutes of Health and the Association for Assessment and Accreditation of Laboratory Animal Care International.

### Mouse lines

*Adamtsl3*^*flox/flox*^ mice with conditional alleles carrying loxP sites in exons 2 and 10 were generated using CRISPR/cas9 (Cyagen, USA) in BL6 background. *Adamtsl3* knock-out mice were generated through lateral ventricle P0 injection of AAV8-hSyn1-RFP-Cre (or AAV8-hSyn1-RFP control), AAV8-hSyn1-ERT2-Cre, AAV2/6-CaMKIIα-ERT2-Cre, and AAV8-hDlx-ERT2-Cre as previously described [25, 26]. AAV injections were assigned at random to littermates from homozygous breeding. Both male and female mice were used for all in vivo experiments, and sex was balanced across experimental groups. Cre recombinase activity from ERT2-Cre was induced at 8 weeks by five consecutive days of tamoxifen injection (i.p) (dissolved in corn oil, Sigma T5648; 1 mg/ day). Control animals were injected with vehicle alone. Animals receiving tamoxifen (TAM+) were assigned at random. Adult PV-specific knock-out mice were generated through stereotactic injection of AAV9/2-S5E3-RFP-Cre (or AAV9/2-S5E3-RFP control) in V1 and animals receiving Cre or control vectors were assigned at random. All recombinant AAVs were produced by the Viral Vector Core at the University of Zürich, except for AAV2/6-CaMKIIα-ERT2-Cre, which was obtained from Vector Biolabs (#2014-1208). PV-Cre mice (B6;129P2-Pvalbtm1(cre)Arbr/J, Jackson ID 008069) were generously provided by Takao Hensch, Harvard University. Adamtsl3-PV-Cre mouse line was generated by crossing female, heterozygous PV-Cre mice with *Adamtsl3*^*flox/flox*^ mice. All mouse lines were fed with water and food under ad libitum conditions and were housed in a 12 h light/dark cycle. None of the mouse strains used in this study showed any signs of gross abnormalities. The sample size was chosen to ensure adequate power to detect the pre-specified effect size based on the power calculation for a simple two-group comparison.

Animals were allocated to experimental groups using a randomized assignment procedure to minimize selection bias. Randomization was performed using a computer-generated random sequence, ensuring that each animal had an equal probability of being assigned to any group. Where applicable, allocation was stratified to balance key variables (e.g., sex, or age) across groups. Samples were processed in a randomized order, and investigators were blinded to group allocation during data acquisition and analysis whenever possible.

Data variability within each group was assessed and is reported as mean ± standard error of the mean (SEM) (or standard deviation, as appropriate). Estimates of variation were derived from independent biological replicates (n = number of animals), and are presented in the figures and Results sections. The assumption of homogeneity of variance was evaluated prior to statistical testing (e.g., using Levene’s test or an equivalent test). Variances were comparable between groups, justifying the use of parametric statistical tests. Where unequal variances were detected, appropriate corrections (e.g., Welch’s correction) were applied.

### Immunohistochemistry

All mice were anesthetized (Nembutal; 50 mg/kg i.p.) and perfused with ice-cold artificial cerebrospinal fluid (ACSF; 125 mM NaCl, 2.5 mM KCl, 1.25 mM NaH2PO4, 26 mM NaHCO3, 25 mM D-glucose, 2.5 mM CaCl2, and 2 mM MgCl2), which was oxygenated (95% O2, 5% CO2) for 15 min. Brains were immediately fixed in 4% PFA for 90 min at 4 °C. After rinsing in PBS, brains were incubated in 30% sucrose (in PBS) at 4 °C overnight for cryo-protection. The frozen brains were cut into 40 µm thick coronal sections using a sliding microtome for immunohistochemistry and stored in the anti-freeze buffer. Relevant sections were selected and after 3 times 10 min washes in Tris-Triton Solution (50 mM Tris, 150 mM NaCl, 0.05% Triton X-100, pH 7.4), sections were incubated in primary antibody solution (50 mM Tris, 150 mM NaCl, 0.4% Triton X-100, 2% normal goat serum, pH 7.4) at 4 °C overnight. Primary antibodies are listed in **Key Resources Table**. Sections were washed 3 x 10 min in Tris-Triton Solution and incubated in secondary antibody solution (50 mM Tris, 150 mM NaCl, 0.05% Triton X-100, 2% normal goat serum, pH 7.4) for 30 min at room temperature with secondary antibodies raised in goat. All secondary antibodies used were diluted 1:500. Antibodies conjugated to AlexaFluor-488, AlexaFluor-594 and AlexaFluor-647 were purchased from Invitrogen. Sections were washed 3 times for 10 min in Tris-Triton Solution and mounted on gelatin-coated slides using Fluorescence Mounting Medium (Dako).

### Image acquisition and fluorescence intensity quantification

Analyses were performed on coronal sections spanning AP + 0.64 to −4.55 mm relative to bregma, encompassing the full V1 extent. Layer 2/3 V1 fields of view (FOVs) were identified in both hemispheres using the Allen Brain Atlas. Within each FOV, PV+ interneurons were randomly selected based solely on PV immunoreactivity; the remaining channels (WFA/PGC/Tenascin/aMMP9/8-oxo-dG/OTX2) were not viewed during selection to blind the analyst to marker intensity. For AAV-based experiments, AAV8 and AAV2/6 spread was evaluated using a 10× objective, and mice exhibiting inadequate viral transduction/coverage were excluded from subsequent morphological analyses. Z-stack images were acquired for all sections using near–super-resolution Airyscan confocal microscopy (LSM 800 with Airyscan, Carl Zeiss). Images were collected with a 40× oil-immersion objective (NA 1.4) at a pixel size of 112 nm², with 6 optical sections acquired at 0.5 µm intervals. Laser power and detector gain/offset were set using the range indicator to ensure that no channel contained saturated pixels. All imaging parameters were kept constant between groups. Fluorescence intensity and puncta density was quantified from maximum-intensity projections in Fiji/ImageJ using a custom Python-based script (https://github.com/dcolam/Cluster-Analysis-Plugin) implemented as an openly available Fiji/ImageJ plugin [79, 80]. In brief, for puncta quantification, images were processed by generating an automated ROI/mask for PV+ interneuron somata based on PV immunolabeling. Puncta were then detected and counted within the PV mask to quantify somatic Syt2 puncta on PV+ neurons, and puncta in the surrounding neuropil were quantified using the inverse mask (excluding PV+ somata) with the same detection parameters. To reduce variability, images were captured from 3 sections per mouse, and a minimum of 3 mice were analyzed per genotype. For each mouse, 15 PV+ interneurons per section were quantified across 3 sections. Representative images were processed in Fiji/ImageJ. Statistical analyses were performed in Prism (GraphPad), with n ≥ 3 mice per group, and are provided in the figure legends.

### Plasmids and Adamtsl3 deletion mutants

pBP84-eGFP-Adamtsl3 plasmid was generated and generously provided by Berangere Pinan Lucarre, University of Lyon. pCMV6-myc-MMP9 and pCMV6-FLAG-TIMP-1 plasmid were purchased from Origene. MMP9 point mutant G100L was generated in-house using site-directed mutagenesis, and *Adamtsl3* deletion mutants were generated by VectorBuilder Inc using site-directed mutagenesis. All expression constructs used were confirmed and checked for protein expression before HEK293T cell/primary neuron transfection.

### Immunoprecipitation and western blot

Immunoprecipitation and Western blot protocols were performed using whole cell lysate or serum-free media from HEK293T cells (ATCC, female donor origin) or mouse brain tissue in EBC lysis buffer (50 mM Tris, pH 7.6, 150 mM NaCl, 1% Triton X-100 containing CompleteMini Protease Inhibitor (Roche), and phosphatase inhibitor mixture 1 and 3 (Sigma Aldrich). For immunoprecipitation, 1 µg of anti-myc antibody (**Key Resources Table**) was used to precipitate myc-tagged MMP9 from transfected HEK293T cell lysate and serum-free, conditioned media for 90 min at 4 °C on a rotor. Complexes were collected by incubating lysates with 25 µl of Pierce Protein A/G Ultralink Resin (Thermo, Cat no. 53132) for 45 min at 4 °C on a rotor. Complexes were centrifuged and washed three times in ice cold EBC buffer to remove nonspecific interactions, followed by denaturation in 2xSDS loading buffer at 90 °C for 5 min. The immunoprecipitated complexes were analyzed using Western blot.

Retinas were isolated through a slit in the cornea and western blot analysis of mouse retina was performed following sonication in Tris-HCl (100 mM, pH 7.5). Western blot analysis of mouse brain was performed following homogenization and 1 h incubation on ice in EBC lysis buffer. Lysates were centrifuged at 20,000 RPM for 30 min at 4 °C, and supernatants were stored at −80 °C. Samples were run on Tris-glycine polyacrylamide gels and proteins were transferred to PVDF membranes. Primary antibodies (see **Key Resources Table**) were incubated in Tris-buffered saline with 0.05% Tween 20 (TBST), including 5% Western Blocking solution (Roche) overnight at 4 °C. Membranes were washed 5 × 5 min in TBST. HRP-coupled donkey secondary antibodies (1:30,000) and fluorescent-coupled donkey secondary antibodies (1:20,000) were incubated for 30 min at room temperature, and membranes were washed again 5 times 5 min in TBST. Fluorescent signals were captured using the Odyssey® CLx Imager. Super Signal West PICO Chemiluminescent substrate (Thermo Fisher Scientific) was applied to visualize HRP labelled antibodies and developed using the FUJIFILM Luminescent Image Analyzer LAS-1000 plus & Intelligent Dark Box II (Fujifilm). Images were analyzed using ImageJ and statistical tests were performed using Prism software (GraphPad) using a minimum of 3 mice.

### Primary neuron culture transfection and immunofluorescence

Primary rat embryonic hippocampal neuron culture preparation and transfection were done as described previously [81]. Rat cultures were used because lipofection is more efficient and consistent than in mouse primary neurons. Cells were transfected after 7 DIV with 1 μg of myc-MMP9 using lipofectamine 2000 (Thermo Fisher Scientific) and magnetofection beads (1:10 diluted, CombiMag, OZ Bioscience). After 15 min of incubation at room temperature, the plasmid-lipofectamine-magnetofection mix was added to cover slips / culture well. Culture plates were placed on top of magnetic plates during the 45 min transfection in the 37 °C incubator. The coverslips were transferred to conditioned media and processed for immunofluorescence at 14 DIV. Coverslips were fixed in 4% paraformaldehyde for 10 min at room temperature, permeabilized with 0.1% Triton X-100 in PBS containing 10% normal goat serum, and stained using anti-myc and anti-Adamtsl3 antibody for 90 min at room temperature. Goat anti-Alexa 488 (Molecular Probes) and goat anti-Cy3 (Jackson Immunoresearch) were used to label the proteins for immunofluorescence. All secondary antibodies used were diluted at 1:500. Coverslips were mounted on glass slides using a fluorescence mounting medium (DAKO).

### Fluorescence in situ hybridization (ISH)

Brain tissue (fresh, not fixed) was embedded in OCT compound (Tissue-Tek, Torrance, CA, USA) and immediately flash frozen on dry ice. The embedded tissue was then sectioned at 20 μm on a cryostat, mounted directly onto Superfrost Plus slides (Thermo Fisher Scientific, Waltham, MA, USA). Triple fluorescence ISH for Adamtsl3, Parvalbumin and CamKIIα was conducted on sections obtained from PV-Cre (control) and PV-Adamtsl3-KO mice using the probes Adamtsl3 (ACDbio, 474891) and Parvalbumin (ACDbio, 421931). All incubations were performed at 40 °C in a hybridization oven (HybEZ, ACD, Hayward, CA, USA) using RNAscope Fluorescent Multiplex Detection Kit (ACDbio, Cat no. 320851) according to the manufacturer’s instructions for fresh-frozen brain tissue. Images were taken using a 40x objective with a numerical aperture of 1.4, and pixel size of 112 nm^2^. All imaging parameters were kept constant between groups and representative example images were processed using ImageJ.

### Slice Preparation and Electrophysiological Recordings

Acute brain slices were prepared from adult (P ~ 120–210) mice with 2–7 neurons recorded per animal. The brain was extracted after terminal anaesthesia with isoflurane and immersed in ice-cold slicing solution composed of (mM): 130 K-gluconate,15 KCl, 0.05 EGTA, 20 HEPES,4 Na pyruvate, 25 glucose and 2 kynurenic acid, pH 7.4, bubbled with 5% CO_2_ and 95% O_2_. 350 μm coronal sections containing the visual cortex were cut using a Leica VT1200S vibroslicer. The slicing solution was exchanged at 37 °C for 60 min with a recording solution containing (mM): 125 NaCl, 2.5 KCl, 2 CaCl_2_, 1 MgCl_2_, 1.25 NaH_2_PO_4_, 26 NaHCO_3_, 2 kynurenic acid, and 25 glucose, pH 7.4. Slices were equilibrated at room temperature for at least 1 hr before recording. Whole-cell recordings of inhibitory postsynaptic currents were carried out using patch electrodes of resistances 3 – 5 MΩ filled with an internal solution containing (mM): 120 CsCl, 1 MgCl_2_, 11 EGTA, 30 KOH, 10 HEPES, 1 CaCl_2_, and 2 K_2_ATP; pH 7.3. Slices were superfused with the recording solution, and cells were at a holding potential of −60 mV with optimized series resistance (Rs <10 MΩ) and whole-cell membrane capacitance compensation. Changes of Rs greater than 10% during the experiment resulted in the recording being excluded from analysis. IPSCs were analyzed using WinEDR and WinWCP (John Dempster, University of Strathclyde, UK), and the frequency was calculated over 60-second epochs of recording. IPSC amplitude distributions were constructed by using the first hundred IPSC amplitudes per cell. IPSC kinetics were measured from uncontaminated events.

### Adult ocular dominance plasticity

P60-P65 *Adamtsl3*^*flox/flox*^ mice of both sexes received stereotaxic injections of either AAV9/2-S5E3-RFP-Cre or control AAV9/2-S5E3-RFP, combined with implantation of tapered tungsten microelectrodes (300–500 MΩ) into layer 4 of the binocular zone, as previously described [53, 82]. Following surgery, a 21-day period was allowed for viral expression and *Adamtsl3* deletion before experimental manipulations. Habituation began two days prior to the initial visual evoked potential (VEP) measurements. On the first day, mice were acclimated to restraint while viewing a gray screen binocularly for 30 min. On the following day, animals experienced 15 min of monocular viewing per eye, achieved by covering the non-stimulated eye with an opaque paddle. For VEP acquisition, animals were presented with sinusoidal gratings (0.2 cycles/degree, 100% contrast) that reversed phase at 2 Hz, following standard procedures [53, 82]. A novel stimulus orientation differing by at least 30° from prior sessions was used each day. Signals were processed using custom C++ and MATLAB scripts. Monocular deprivation (MD) was induced at P79-82, 1–3 h after recording baseline PreMD VEPs. Animals were anesthetized with isoflurane (1–3% in oxygen) and kept on a heating pad throughout the procedure. One eye was sutured closed with 1–2 mattress stitches (Prolene 6-0, Ethicon). After four days, sutures were removed under anaesthesia, eyes were rinsed with sterile drops, and Post MD VEPs were collected ≥2 h later to ensure recovery. Eyes were examined pre- and post-MD to confirm ocular health before data collection, and only VEP recordings with stable baselines and clear signal-to-noise ratios were included in the analysis.

### Gelatinase zymography assay

MMP9 activity was assessed using the gelatin zymography protocol by Abcam. HEK293T cells (ATCC, female donor origin) were grown to 50% confluency before transfection of eGFP-Adamtsl3, eGFP-Adamtsl3 deletion mutants, FLAG-TIMP-1, myc-MMP9 expression plasmids using standard PEI-based transfection at a ratio of 1 µg plasmid to 4 µg PEI. At 70–80% confluency, cells were washed and continued to grow cells in FBS-free media. 24–48 h later, conditioned media was collected and concentrated 10x. Relevant samples were prepared and combined in 5x non-reducing sample buffer at 10 µg/ml per protein sample and run on a 7.5% acrylamide gel containing gelatin (4 mg/ml). Gels were washed to remove SDS (2.5% Triton X-100, 50 mM Tris-HCl pH 7.5, 5 mM CaCl_2_, 1 µM ZnCl_2_) and incubated with incubation buffer (1% Triton x 100, 50 mM Tris HCl pH 7.5, 50 mM CaCl_2_) for 24 h at 37 °C. Gels were stained with Coomassie Blue for 1 h at RT and destained until clear bands were detected. For loading control, samples were run on Tris-glycine polyacrylamide gels and analyzed by Western blot as described above. Images were captured using FUJIFILM Luminescent Image Analyzer LAS-1000 plus & Intelligent Dark Box II (Fujifilm) analyzed using ImageJ using a minimum of 3 samples/group. Statistical tests were performed using Prism software (GraphPad).

### SB-3CT administration

SB-3CT (APExBIO, Cat no. B4792) was dissolved in 25% DMSO, 65% PEG 400 and 10% water. Vehicle control injections received the same solvent mixture without SB-3CT. After filtration, animals were injected i.p. at 25 mg/kg for four consecutive days. At day 5, mice were anesthetized and perfused with ACSF as described above.

### Materials availability

All reagents generated in this study will be made available from the lead contact under suitable materials transfer agreements.

## Supplementary information

Supplemental Figures

## Data Availability

All relevant data from the manuscript are available upon specific request to Prof. Dr. Shiva K. Tyagarajan, University of Coimbra. The *Adamtsl3*^flox/flox^ mouse line and Adamtsl3 antibody will be made available under standard institutional MTA.

## References

[1] Hensch TK. Critical period plasticity in local cortical circuits. Nat Rev Neurosci. 2005;6:877–88. 10.1038/nrn1787 16261181

[2] Zimmermann DR, Dours-Zimmermann MT. Extracellular matrix of the central nervous system: from neglect to challenge. Histochem Cell Biol. 2008;130:635–53. 10.1007/s00418-008-0485-9 18696101

[3] Tau GZ, Peterson BS. Normal development of brain circuits. Neuropsychopharmacology. 2010;35:147–68. 10.1038/npp.2009.115 PMC3055433 19794405

[4] Reh RK, Dias BG, Nelson CA 3rd, Kaufer D, Werker JF, Kolb B, et al. Critical period regulation across multiple timescales. Proc Natl Acad Sci USA. 2020;117:23242–51. 10.1073/pnas.1820836117 PMC7519216 32503914

[5] Zhang ZJ, Reynolds GP. A selective decrease in the relative density of parvalbumin-immunoreactive neurons in the hippocampus in schizophrenia. Schizophrenia Res. 2002;55:1–10. 10.1016/s0920-9964(01)00188-8 11955958

[6] Marin O. Interneuron dysfunction in psychiatric disorders. Nature. 2012;13:107–20. 10.1038/nrn3155 22251963

[7] Wen TH, Afroz S, Reinhard SM, Palacios AR, Tapia K, Binder DK, et al. Genetic reduction of matrix Metalloproteinase-9 promotes formation of perineuronal nets around parvalbumin-expressing interneurons and normalizes auditory cortex responses in developing Fmr1 knock-out mice. Cereb Cortex. 2018;28:3951–64. 10.1093/cercor/bhx258 PMC6188540 29040407

[8] Ferguson BR, Gao WJ. PV interneurons: critical regulators of E/I balance for prefrontal cortex-dependent behavior and psychiatric disorders. Front Neural Circuits. 2018;12:37. 10.3389/fncir.2018.00037 PMC5964203 29867371

[9] Wen TH, Binder DK, Ethell IM, Razak KA. The perineuronal ‘Safety’ Net? Perineuronal net abnormalities in neurological disorders. Front Mol Neurosci. 2018;11:270. 10.3389/fnmol.2018.00270 PMC6085424 30123106

[10] Sorg BA, Berretta S, Blacktop JM, Fawcett JW, Kitagawa H, Kwok JC, et al. Casting a wide net: role of perineuronal nets in neural plasticity. J Neurosci. 2016;36:11459–68. 10.1523/JNEUROSCI.2351-16.2016 PMC5125213 27911749

[11] Hall NG, Klenotic P, Anand-Apte B, Apte SS. ADAMTSL-3/punctin-2, a novel glycoprotein in extracellular matrix related to the ADAMTS family of metalloproteases. Matrix Biol. 2003;22:501–10. 10.1016/s0945-053x(03)00075-1 14667842

[12] Kelwick R, Desanlis I, Wheeler GN, Edwards DR. The ADAMTS (A Disintegrin and Metalloproteinase with Thrombospondin motifs) family. Genome Biol. 2015;16:113. 10.1186/s13059-015-0676-3 PMC4448532 26025392

[13] Dubail J, Apte SS. Insights on ADAMTS proteases and ADAMTS-like proteins from mammalian genetics. Matrix Biol. 2015;44–46:24–37. 10.1016/j.matbio.2015.03.001 25770910

[14] Need AC, Ge D, Weale ME, Maia J, Feng S, Heinzen EL, et al. A genome-wide investigation of SNPs and CNVs in schizophrenia. PLoS Genet. 2009;5:e1000373. 10.1371/journal.pgen.1000373 PMC2631150 19197363

[15] Dow DJ, Huxley-Jones J, Hall JM, Francks C, Maycox PR, Kew JNC, et al. ADAMTSL3 as a candidate gene for schizophrenia: gene sequencing and ultra-high density association analysis by imputation. Schizophr Res. 2011;127:28–34. 10.1016/j.schres.2010.12.009 21239144

[16] Oliveira-Silva P, Jurgilas PB, Trindade P, Campello-Costa P, Perales J, Savino W, et al. Matrix metalloproteinase-9 is involved in the development and plasticity of retinotectal projections in rats. Neuroimmunomodulation. 2007;14:144–9. 10.1159/000110638 18073506

[17] Egeblad M, Werb Z. New functions for the matrix metalloproteinases in cancer progression. Nat Rev Cancer. 2002;2:161–74. 10.1038/nrc745 11990853

[18] Beroun A, Mitra S, Michaluk P, Pijet B, Stefaniuk M, Kaczmarek L. MMPs in learning and memory and neuropsychiatric disorders. Cell Mol Life Sci. 2019;76:3207–28. 10.1007/s00018-019-03180-8 PMC6647627 31172215

[19] Ethell IM, Ethell DW. Matrix metalloproteinases in brain development and remodeling: synaptic functions and targets. J Neurosci Res. 2007;85:2813–23. 10.1002/jnr.21273 17387691

[20] Cramer TML, Pinan-Lucarre B, Cavaccini A, Damilou A, Tsai YC, Bhat MA, et al. Adamtsl3 mediates DCC signaling to selectively promote GABAergic synapse function. Cell Rep. 2023;42:112947. 10.1016/j.celrep.2023.112947 37572323

[21] Hensch TK. Critical period regulation. Annu Rev Neurosci. 2004;27:549–79. 10.1146/annurev.neuro.27.070203.144327 15217343

[22] Guttenplan KA, Weigel MK, Adler DI, Couthouis J, Liddelow SA, Gitler AD, et al. Knockout of reactive astrocyte activating factors slows disease progression in an ALS mouse model. Nat Commun. 2020;11:3753. 10.1038/s41467-020-17514-9 PMC7385161 32719333

[23] Hilbig H, Bidmon H-J, Blohm U, Zilles K. Wisteria floribunda agglutinin labeling patterns in the human cortex: a tool for revealing areal borders and subdivisions in parallel with immunocytochemistry. Anat Embryol. 2001;203:45–52. 10.1007/s004290000135 11195088

[24] Ueno H, Fujii K, Suemitsu S, Murakami S, Kitamura N, Wani K, et al. Expression of aggrecan components in perineuronal nets in the mouse cerebral cortex. IBRO Rep. 2018;4:22–37. 10.1016/j.ibror.2018.01.002 PMC6084874 30135949

[25] Cramer TML, Abegg A, Tyagarajan SK. Protocol for in vivo analysis of pre- and post-synaptic protein function in mice. STAR Protoc. 2024;5:103117. 10.1016/j.xpro.2024.103117 PMC11193045 38857153

[26] Kim JY, Grunke SD, Levites Y, Golde TE, Jankowsky JL. Intracerebroventricular viral injection of the neonatal mouse brain for persistent and widespread neuronal transduction. J Vis Exp. 2014;91:51863. 10.3791/51863 PMC4199253 25286085

[27] Hage TA, Bosma-Moody A, Baker CA, Kratz MB, Campagnola L, Jarsky T, et al. Synaptic connectivity to L2/3 of primary visual cortex measured by two-photon optogenetic stimulation. Elife. 2022;11:e71103. 10.7554/eLife.71103 PMC8824465 35060903

[28] Jahn HM, Kasakow CV, Helfer A, Michely J, Verkhratsky A, Maurer HH, et al. Refined protocols of tamoxifen injection for inducible DNA recombination in mouse astroglia. Sci Rep. 2018;8:5913. 10.1038/s41598-018-24085-9 PMC5897555 29651133

[29] Shcholok T, Eftekharpour E. Cre-recombinase systems for induction of neuron-specific knockout models: a guide for biomedical researchers. Neural Regen Res. 2023;18:273–9. 10.4103/1673-5374.346541 PMC9396489 35900402

[30] Dimidschstein J, Chen Q, Tremblay R, Rogers SL, Saldi GA, Guo L, et al. A viral strategy for targeting and manipulating interneurons across vertebrate species. Nat Neurosci. 2016;19:1743–9. 10.1038/nn.4430 PMC5348112 27798629

[31] Miyata S, Nadanaka S, Igarashi M, Kitagawa H. Structural variation of chondroitin sulfate chains contributes to the molecular heterogeneity of perineuronal nets. Front Integr Neurosci. 2018;12:3. 10.3389/fnint.2018.00003 PMC5801575 29456495

[32] Sugiyama S, Di Nardo AA, Aizawa S, Matsuo I, Volovitch M, Prochiantz A, et al. Experience-dependent transfer of Otx2 homeoprotein into the visual cortex activates postnatal plasticity. Cell. 2008;134:508–20. 10.1016/j.cell.2008.05.054 18692473

[33] Takesian AE, Hensch TK. Balancing plasticity/stability across brain development. Prog Brain Res. 2013;207:3–34. 10.1016/B978-0-444-63327-9.00001-1 24309249

[34] Beurdeley MSJ, Lee HHC, Sugiyama S, Bernard C, Di Nardo AA, Hensch TK, et al. Otx2 binding to perineuronal nets persistently regulates plasticity in the mature visual cortex. J Neurosci. 2012;32:9429–37. 10.1523/JNEUROSCI.0394-12.2012 PMC3419577 22764251

[35] Cabungcal JH, Steullet P, Morishita H, Kraftsik R, Cuenod M, Hensch TK, et al. Perineuronal nets protect fast-spiking interneurons against oxidative stress. Proc Natl Acad Sci USA. 2013;110:9130–5. 10.1073/pnas.1300454110 PMC3670388 23671099

[36] Gorini F, Scala G, Cooke MS, Majello B, Amente S. Towards a comprehensive view of 8-oxo-7,8-dihydro-2′-deoxyguanosine: highlighting the intertwined roles of DNA damage and epigenetics in genomic instability. DNA Repair. 2021;97:103027. 10.1016/j.dnarep.2020.103027 PMC7926032 33285475

[37] Valavanidis A, Vlachogianni T, Fiotakis C. 8-hydroxy-2' -deoxyguanosine (8-OHdG): A critical biomarker of oxidative stress and carcinogenesis. J Environ Sci Health C Environ Carcinog Ecotoxicol Rev. 2009;27:120–39. 10.1080/10590500902885684 19412858

[38] Christensen J, Shastri VP. Matrix-metalloproteinase-9 is cleaved and activated by cathepsin K. BMC Res Notes. 2015;8:322. 10.1186/s13104-015-1284-8 PMC4518881 26219353

[39] Gough PJ, Gomez IG, Wille PT, Raines EW. Macrophage expression of active MMP-9 induces acute plaque disruption in apoE-deficient mice. J Clin Invest. 2006;116:59–69. 10.1172/JCI25074 PMC1319218 16374516

[40] Roderfeld M, Graf J, Giese B, Salguero-Palacios R, Tschuschner A, Müller-Newen G, et al. Latent MMP-9 is bound to TIMP-1 before secretion. Biol Chem. 2007;388:1227–34. 10.1515/BC.2007.123 17976016

[41] Meisel JE, Chang M. Selective small-molecule inhibitors as chemical tools to define the roles of matrix metalloproteinases in disease. Biochim Biophys Acta Mol Cell Res. 2017;1864:2001–14. 10.1016/j.bbamcr.2017.04.011 28435009

[42] Gooyit M, Suckow MA, Schroeder VA, Wolter WR, Mobashery S, Chang M. Selective gelatinase inhibitor neuroprotective agents cross the blood-brain barrier. ACS Chem Neurosci. 2012;3:730–6. 10.1021/cn300062w PMC3474282 23077716

[43] Lensjø KK, Lepperod ME, Dick G, Hafting T, Fyhn M. Removal of perineuronal nets unlocks juvenile plasticity through network mechanisms of decreased inhibition and increased gamma activity. J Neurosci. 2017;37:1269–83. 10.1523/JNEUROSCI.2504-16.2016 PMC6596863 28039374

[44] Christensen AC, Lensjo KK, Lepperod ME, Dragly SA, Sutterud H, Blackstad JS, et al. Perineuronal nets stabilize the grid cell network. Nat Commun. 2021;12:253. 10.1038/s41467-020-20241-w PMC7801665 33431847

[45] Wingert JC, Sorg BA. Impact of perineuronal nets on electrophysiology of parvalbumin interneurons, principal neurons, and brain oscillations: a review. Front Synaptic Neurosci. 2021;13:673210. 10.3389/fnsyn.2021.673210 PMC8141737 34040511

[46] Sommeijer JP, Levelt CN. Synaptotagmin-2 is a reliable marker for parvalbumin positive inhibitory boutons in the mouse visual cortex. PLoS one. 2012;7:e35323. 10.1371/journal.pone.0035323 PMC3335159 22539967

[47] Sigal YM, Bae H, Bogart LJ, Hensch TK, Zhuang X. Structural maturation of cortical perineuronal nets and their perforating synapses revealed by superresolution imaging. Proc Natl Acad Sci USA. 2019;116:7071–6. 10.1073/pnas.1817222116 PMC6452715 30890637

[48] Liu L, Zhang Y, Ju J. Removal of perineuronal nets leads to altered neuronal excitability and synaptic transmission in the visual cortex with distinct time courses. Neurosci Lett. 2022;785:136763. 10.1016/j.neulet.2022.136763 35760385

[49] Liu L, Zhang Y, Men S, Li X, Hou S-T, Ju J. Elimination of perineuronal nets in CA1 disrupts GABA release and long‐term contextual fear memory retention. Hippocampus. 2023;33:862–71. 10.1002/hipo.23503 36709413

[50] Federer F, Balsor J, Ingold A, Babcock DP, Dimidschstein J, Angelucci A. Laminar specificity and coverage of viral-mediated gene expression restricted to GABAergic interneurons and their parvalbumin subclass in marmoset primary visual cortex. Elife. 2024;13:RP97673. 10.7554/eLife.97673 PMC11412690 39297605

[51] Fawcett JW, Oohashi T, Pizzorusso T. The roles of perineuronal nets and the perinodal extracellular matrix in neuronal function. Nat Rev Neurosci. 2019;20:451–65. 10.1038/s41583-019-0196-3 31263252

[52] Frenkel MY, Bear MF. How monocular deprivation shifts ocular dominance in visual cortex of young mice. Neuron. 2004;44:917–23. 10.1016/j.neuron.2004.12.003 15603735

[53] Sawtell NB, Frenkel MY, Philpot BD, Nakazawa K, Tonegawa S, Bear MF. NMDA receptor-dependent ocular dominance plasticity in adult visual cortex. Neuron. 2003;38:977–85. 10.1016/s0896-6273(03)00323-4 12818182

[54] Ye Q, Miao QL. Experience-dependent development of perineuronal nets and chondroitin sulfate proteoglycan receptors in mouse visual cortex. Matrix Biol. 2013;32:352–63. 10.1016/j.matbio.2013.04.001 23597636

[55] Schizophrenia Working Group of the Psychiatric Genomics C. Biological insights from 108 schizophrenia-associated genetic loci. Nature. 2014;511:421–7. 10.1038/nature13595 PMC4112379 25056061

[56] Trubetskoy V, Pardiñas AF, Qi T, Panagiotaropoulou G, Awasthi S, Bigdeli TB, et al. Mapping genomic loci implicates genes and synaptic biology in schizophrenia. Nature. 2022;604:502–8. 10.1038/s41586-022-04434-5 PMC9392466 35396580

[57] Minns AF, Qi Y, Yamamoto K, Lee K, Ahnstrom J, Santamaria S. The C-terminal domains of ADAMTS1 contain exosites involved in its proteoglycanase activity. J Biol Chem. 2023;299:103048. 10.1016/j.jbc.2023.103048 PMC10033314 36813235

[58] Kashiwagi M, Enghild J, Gendron C, Hughes C, Caterson B, Itoh Y, et al. Altered proteolytic activities of ADAMTS-4 expressed by C-terminal processing*. J Biol Chem. 2004;279:10109–19. 10.1074/jbc.M312123200 14662755

[59] Soejima K, Matsumoto M, Kokame K, Yagi H, Ishizashi H, Maeda H, et al. ADAMTS-13 cysteine-rich/spacer domains are functionally essential for von Willebrand factor cleavage. Blood. 2003;102:3232–7. 10.1182/blood-2003-03-0908 12869506

[60] Bein K, Simons M. Thrombospondin type 1 repeats interact with matrix metalloproteinase 2. Regulation of metalloproteinase activity. J Biol Chem. 2000;275:32167–73. 10.1074/jbc.M003834200 10900205

[61] Collado-Torres L, Burke E, Peterson A, Shin J, Jaffe A. Regional heterogeneity in gene expression, regulation, and coherence in the frontal cortex and hippocampus across development and schizophrenia. Neuron. 2019;103:203–16. 10.1016/j.neuron.2019.05.013 PMC7000204 31174959

[62] Pantazopoulos H, Katsel P, Haroutunian V, Chelini G, Klengel T, Berretta S. Molecular signature of extracellular matrix pathology in schizophrenia. Eur J Neurosci. 2021;53:3960–87. 10.1111/ejn.15009 PMC8359380 33070392

[63] Dwir D, Giangreco B, Xin L, Tenenbaum L, Cabungcal J, Steullet P, et al. MMP9/RAGE pathway overactivation mediates redox dysregulation and neuroinflammation, leading to inhibitory/excitatory imbalance: a reverse translation study in schizophrenia patients. Mol Psychiatry. 2019;25:2889–904. 10.1038/s41380-019-0393-5 PMC7577857 30911107

[64] Murase S, Robertson S, Lantz C, Liu J, Winkowski D, Quinlan E. Chronic monocular deprivation reveals MMP9-dependent and -independent aspects of murine visual system plasticity. Int J Mol Sci. 2022;8:e52503. 10.3390/ijms23052438 PMC8909986 35269580

[65] Michaluk P, Kaczmarek L. Matrix metalloproteinase-9 in glutamate-dependent adult brain function and dysfunction. Cell Death Differ. 2007;14:1255–8. 10.1038/sj.cdd.4402141 17431423

[66] Devienne G, Picaud S, Cohen I, Piquet J, Tricoire L, Testa D, et al. Regulation of perineuronal nets in the adult cortex by the activity of the cortical network. J Neurosci. 2021;41:5779–90. 10.1523/JNEUROSCI.0434-21.2021 PMC8265812 34045309

[67] Katagiri H, Fagiolini M, Hensch TK. Optimization of somatic inhibition at critical period onset in mouse visual cortex. Neuron. 2007;53:805–12. 10.1016/j.neuron.2007.02.026 17359916

[68] Maffei A, Nataraj K, Nelson SB, Turrigiano GG. Potentiation of cortical inhibition by visual deprivation. Nature. 2006;443:81–4. 10.1038/nature05079 16929304

[69] Tewari BP, Chaunsali L, Campbell SL, Patel DC, Goode AE, Sontheimer H. Perineuronal nets decrease membrane capacitance of peritumoral fast spiking interneurons in a model of epilepsy. Nat Commun. 2018;9:4724. 10.1038/s41467-018-07113-0 PMC6226462 30413686

[70] Hanssen K, Grødem S, Fyhn M, Hafting T, Einevoll G, Ness T, et al. Responses in fast-spiking interneuron firing rates to parameter variations associated with degradation of perineuronal nets. J Computational Neurosci. 2023;51:283–98. 10.1007/s10827-023-00849-9 PMC10182141 37058180

[71] Rowlands D, Lensjø K, Dinh T, Yang S, Andrews M, Hafting T, et al. Aggrecan directs extracellular matrix-mediated neuronal plasticity. J Neurosci. 2018;38:10102–13. 10.1523/JNEUROSCI.1122-18.2018 PMC6596198 30282728

[72] Carceller H, Guirado R, Ripolles-Campos E, Teruel-Martí V, Nácher J. Perineuronal Nets regulate the inhibitory perisomatic input onto parvalbumin interneurons and γ activity in the prefrontal cortex. J Neurosci. 2020;40:5008–18. 10.1523/JNEUROSCI.0291-20.2020 PMC7314408 32457072

[73] Faini G, Aguirre A, Landi S, Lamers D, Pizzorusso T, Ratto GM, et al. Perineuronal nets control visual input via thalamic recruitment of cortical PV interneurons. Elife. 2018;7:e41520. 10.7554/eLife.41520 PMC6298774 30561327

[74] Rotstein B, Post Y, Reinhardt M, Lammers K, Buhr A, Heinisch JJ, et al. Distinct domains in the matricellular protein Lonely heart are crucial for cardiac extracellular matrix formation and heart function in Drosophila. J Biol Chem. 2018;293:7864–79. 10.1074/jbc.M117.817940 PMC5961049 29599288

[75] Nardi JB, Martos R, Walden KK, Lampe DJ, Robertson HM. Expression of lacunin, a large multidomain extracellular matrix protein, accompanies morphogenesis of epithelial monolayers in Manduca sexta. Insect Biochem Mol Biol. 1999;29:883–97. 10.1016/s0965-1748(99)00064-8 10528409

[76] Pizzorusso T, Medini P, Berardi N, Chierzi S, Fawcett JW, Maffei L. Reactivation of ocular dominance plasticity in the adult visual cortex. Science. 2002;298:1248–51. 10.1126/science.1072699 12424383

[77] Spolidoro M, Putignano E, Munafo C, Maffei L, Pizzorusso T. Inhibition of matrix metalloproteinases prevents the potentiation of nondeprived-eye responses after monocular deprivation in juvenile rats. Cereb Cortex. 2012;22:725–34. 10.1093/cercor/bhr158 21685398

[78] Maya Vetencourt JF, Sale A, Viegi A, Baroncelli L, De Pasquale R, O’Leary OF, et al. The antidepressant fluoxetine restores plasticity in the adult visual cortex. Science. 2008;320:385–8. 10.1126/science.1150516 18420937

[79] Schindelin J, Arganda-Carreras I, Frise E, Kaynig V, Longair M, Pietzsch T, et al. Fiji: an open-source platform for biological-image analysis. Nat Methods. 2012;9:676–82. 10.1038/nmeth.2019 PMC3855844 22743772

[80] Cramer TML, Tyagarajan SK. Protocol for the culturing of primary hippocampal mouse neurons for functional in vitro studies. STAR Protoc. 2024;5:102991. 10.1016/j.xpro.2024.102991 PMC11017351 38607922

[81] Buerli T, Pellegrino C, Baer K, Lardi-Studler B, Chudotvorova I, Fritschy JM, et al. Efficient transfection of DNA or shRNA vectors into neurons using magnetofection. Nat Protoc. 2007;2:3090–101. 10.1038/nprot.2007.445 18079708

[82] Fong MF, Mitchell DE, Duffy KR, Bear MF. Rapid recovery from the effects of early monocular deprivation is enabled by temporary inactivation of the retinas. Proc Natl Acad Sci USA. 2016;113:14139–44. 10.1073/pnas.1613279113 PMC5150384 27856748

