# Supplementary material for: Schizophrenia-associated glycoprotein Adamtsl3 regulates perineuronal net formation, maintenance, and adult cortical plasticity
Source: Mol Psychiatry. 2026 Jun 11;31(11):6153–66. doi: 10.1038/s41380-026-03672-7 (PMC13668732; doi:10.1038/s41380-026-03672-7)
Supplement: Supplementary file 1 [file 41380_2026_3672_MOESM1_ESM.pdf]

Schizophrenia-Associated Glycoprotein Adamtsl3 Regulates Perineuronal Net Formation, Maintenance, and Adult Cortical Plasticity

Authors: Teresa M. L. Cramer, PhD[2,3,6], Saad B. Hannan, PhD[3], Lucie Petre, MSc[2], Mark F. Bear, PhD[6], Takao K. Hensch, PhD[3,4,5 #], Shiva K. Tyagarajan, PhD[1,2,# *]

# co-senior authors

*Correspondence: shiva.tyagarajan@uc.pt

Supplementary Figure 1. Adamtsl3 is not associated with glial cells in V1. Representative images showing Adamtsl3 (green) co-stained with the astrocyte marker ALDH1L1 (red) or the microglial marker Iba-1 (red) in layer 2/3 of V1 from adult C57/BL6 mice (P84). Scale bar 10 µm.

Supplementary Figure 2. Adamtsl3 influences aggrecan accumulation during PNN maturation and adult maintenance in V1. (A) Illustration of P0 lateral ventricle injection. Right: representative AAV8 infectivity in V1 at P17. Scale bar 150µm. (B) Representative images showing reduction of aggrecan (green) around L2/3 PV+ (magenta) cells in AAV8-hSyn1-RFP-Cre injected animals at P17, P21, P24, and P30. Below: quantification relative to control (n = 3-4, 15 PV+ cells/ animal). Scale bar 10µm (C) Timeline of P0 lateral ventricle injection with AAV8-hSyn1-ERT2-Cre. Right: representative viral infectivity in V1 at P90. Scale bar 100µm. (D) Representative images showing reduction of WFA (green) or aggrecan (green) around L2/3 PV+ (magenta) cells in TAM+ AAV8-hSyn1-ERT2-Cre animals. Scale bar 10µm. Right: quantification relative to TAM- controls (n = 3-4, 15 PV+ cells/ animal). Data is shown as mean ± SD. Student's two-tailed t-test * $P < 0.05$, ** $P < 0.01$.

Supplementary Figure 3. Adamtsl3 influences MMP9 activity. (A) Representative images showing reduction of Otx2 (blue) in L2/3 PV+ cells (magenta) in AAV8-hSyn1-RFP-Cre

injected animals at P17, P24, P30 and P90. Below: quantification relative to control (n = 3-4, 15 PV+ cells/ animal). (B) Western blot for Otx2 in P30 cortical and retinal lysates (n = 4). (C) Representative images showing 8-oxo-dG (green) in PV+ (magenta) cells in control and cre injected animals at P30 and P90. Right: quantification relative to controls (n = 3-4, 15 PV+ cells/ animal). Scale bar 10 μm. Data is shown as mean ± SD. Student's two-tailed t-test, * $P < 0.05$, ** $P < 0.01$, *** $P < 0.001$.

Supplementary Figure 4. Adamtsl3 regulates MMP9 activity via distinct domains. (A) Illustration of full-length Adamtsl3 (WT) and deletion constructs M# 1 – M# 5. (B) Gelatinase zymography of myc-MMP9 alone/with Adamtsl3 WT or deletion constructs (n=4-6; One-way ANOVA, Bonferroni multiple comparison test, $F(7,25) = 37.07$, $p < 0.0001$). Below: representative MMP9 activity bands. (C) Representative images showing an increase in active MMP9 (green) around L2/3 PV+ (magenta) cells in AAV8-hSyn1-RFP-Cre injected animals at P24, P30 and P90. Scale bar 10 μm. Below: quantification relative to control (n = 3-4, 15 PV+ cells/ animal). Data is shown as mean ± SD. Student's two-tailed t-test, * $P < 0.05$, ** $P < 0.01$.

Supplementary Figure 5. PV cell-specific deletion of Adamtsl3 influences PV+ synaptic connectivity. (A) Representative *in situ* hybridization images for PV (magenta) and Adamtsl3 (green) in PV-KO compared to controls. (B) Representative images showing PV (magenta) and Adamtsl3 (green) expression in L2/3 PV+ neurons of PV-KO mice and Ctrl.(B') Representative images showing Adamtsl3 (green) expression in L5 pyramidal neurons of PV-KO mice and Ctrl as an internal control for PV-Cre specificity (preserved expression in non-PV+ neurons). Scale bar 10 μm. (C) Quantification of PV+ cell density in V1 of both genotypes. Scale bar 150 μm. Right: quantification of PV+ cell count (n = 5). (D) Representative images showing Synaptotagmin 2 (Syt2, green) onto PV+ (magenta) cells. Scale bar 10 μm. Right: quantification of puncta density on PV+ cells (n = 5, 15 PV+ cells/ animal). (D') Representative

images showing Synaptotagmin 2 (Syt2, green) expression in L2/3. Scale bar 30 μm. Right: quantification of puncta density (n = 5, 6 images/animal). Data is shown as mean ± SD. Student's two-tailed t-test, * P< 0.05, ** P< 0.01.

Supplementary Figure 6. Adult PV-specific Adamtsl3 deletion reduces PNNs and increases VEP magnitude. (A) Representative AAV9/2-S5E3-RFP-Cre and AAV9/2-S5E3-RFP infectivity in V1 21 days post injection**.** (B) Representative images showing S5E3-RFP or S5E3-RFP-Cre (red) and PV (green) in V1. Right: Quantification of S5E3-RFP$^+$ cells that co-express PV. (C) Representative images showing WFA (green) and PV (magenta) labeling in V1 of S5E3-RFP-Cre–injected animals. Right: quantification of RFP$^+$ cells covered by WFA. Scale bar 150 μm. (D) Experimental timeline. (E) Visual evoked potential (VEP) responses recorded from binocular V1 of AAV9/2-S5E3-RFP-Cre– and AAV9/2-S5E3-RFP–injected animals, 21 days post injection. (E') Averaged VEP traces from control and S5E3-KO animals. Data is shown as mean ± SD. Student's two-tailed t-test, * P< 0.05, ** P< 0.01.

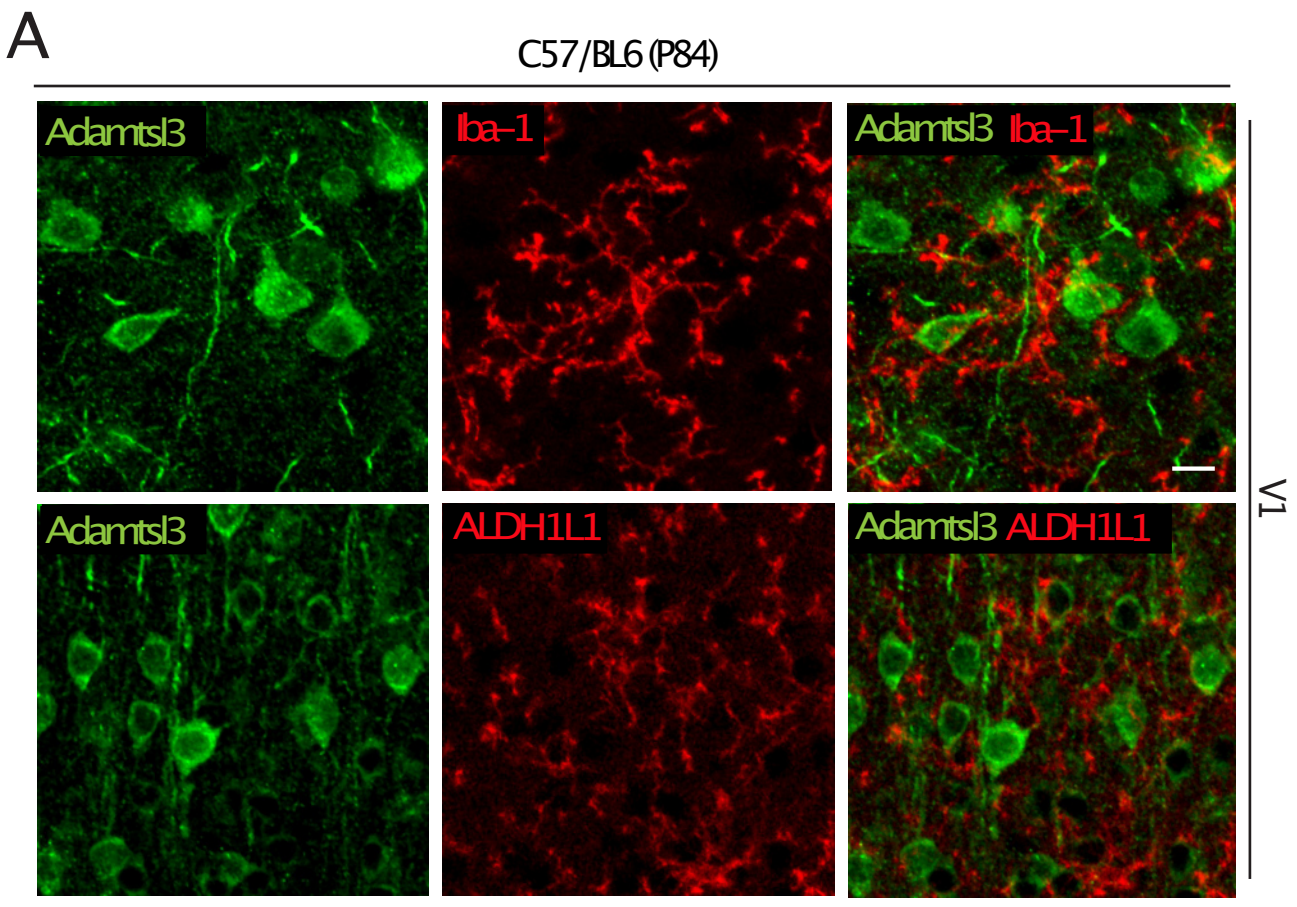

Supplementary Figure 1. Adamtsl3 is not associated with glial cells in V1 area.

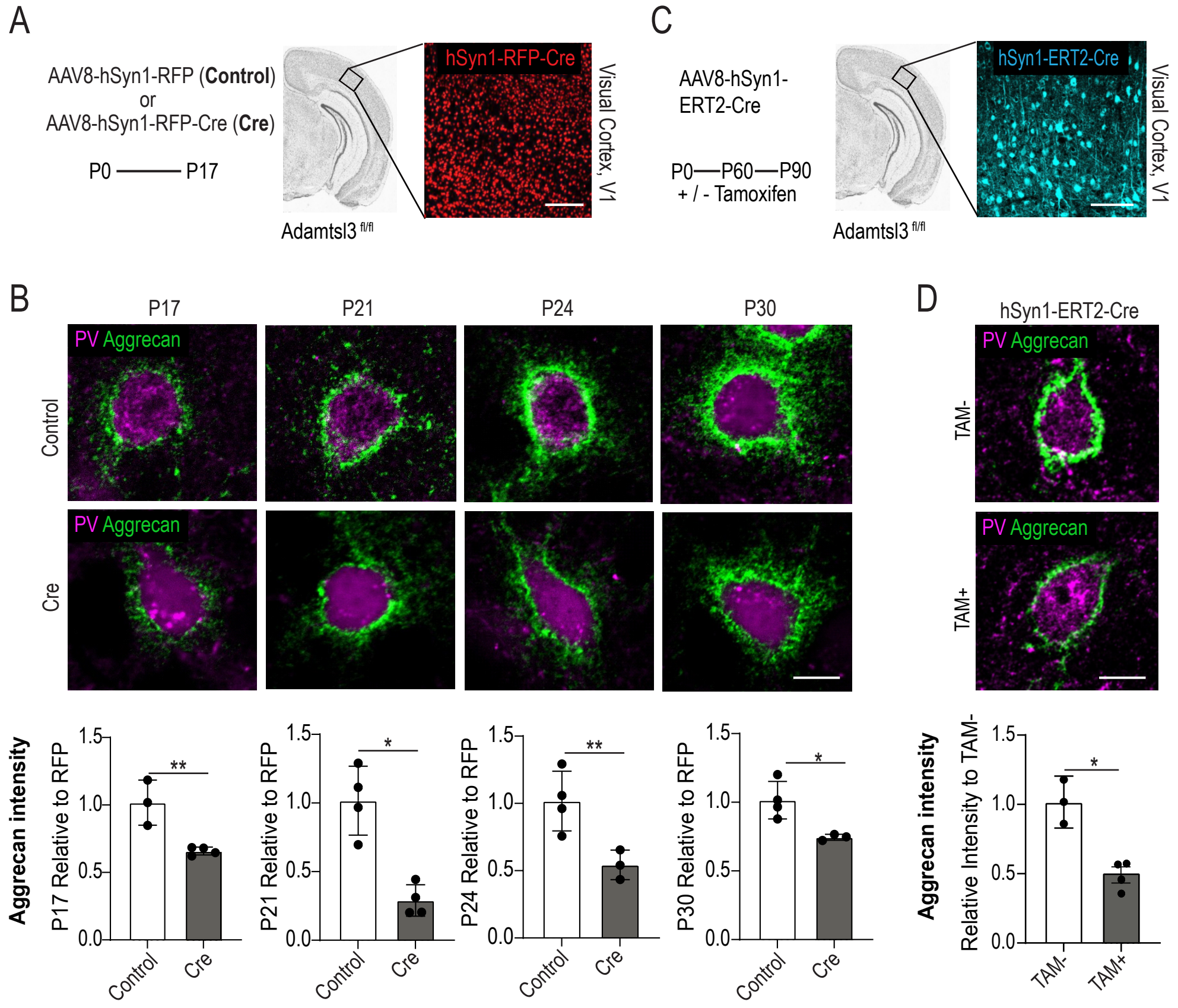

Suppl Figure 2. Adamtsl3 influences aggrecan accumulation during PNN maturation and adult maintenance in V1.

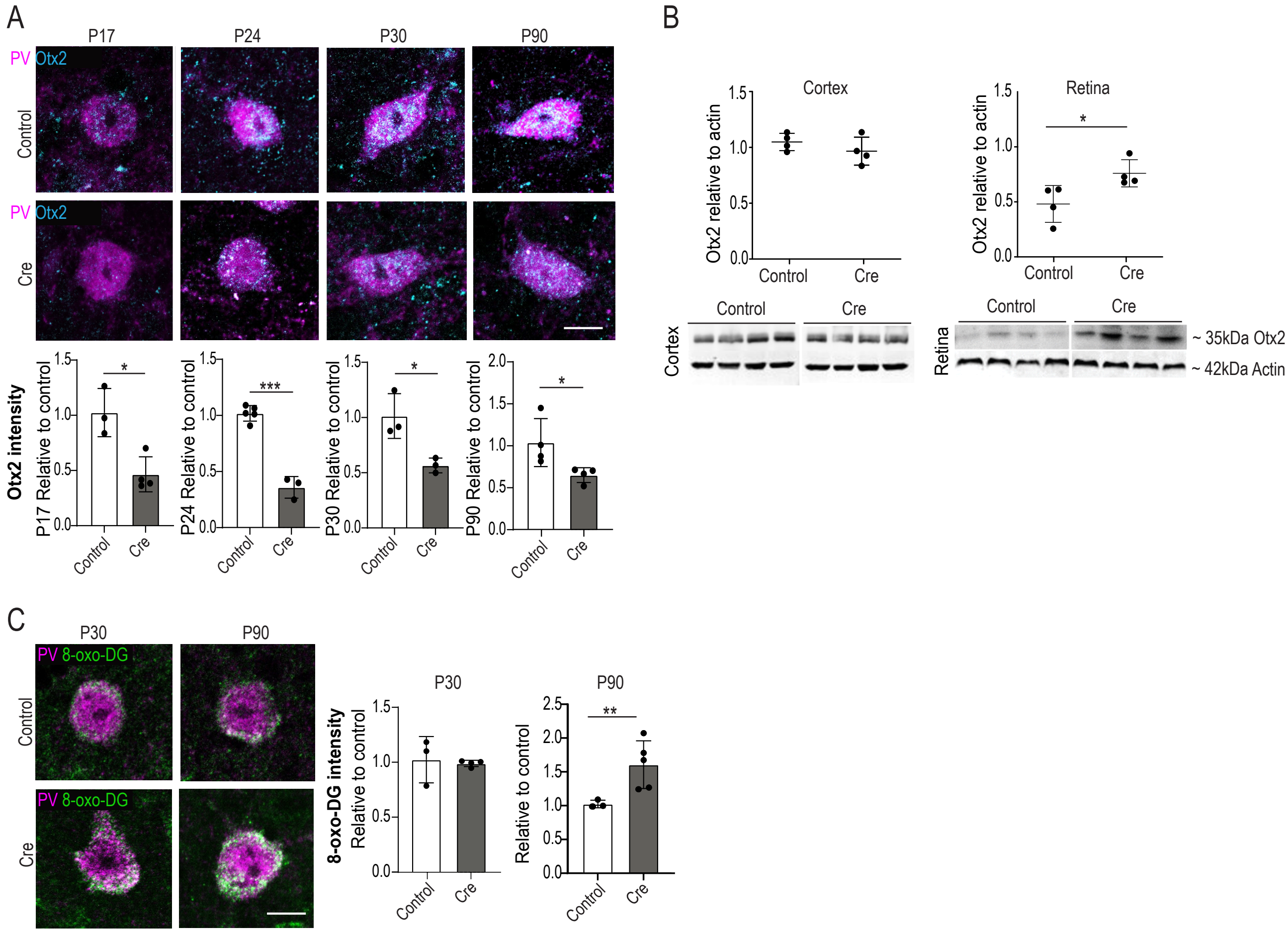

Supplementary Figure 3. Adamtsl3 deletion impairs Otx2 uptake and elevates oxidative stress.

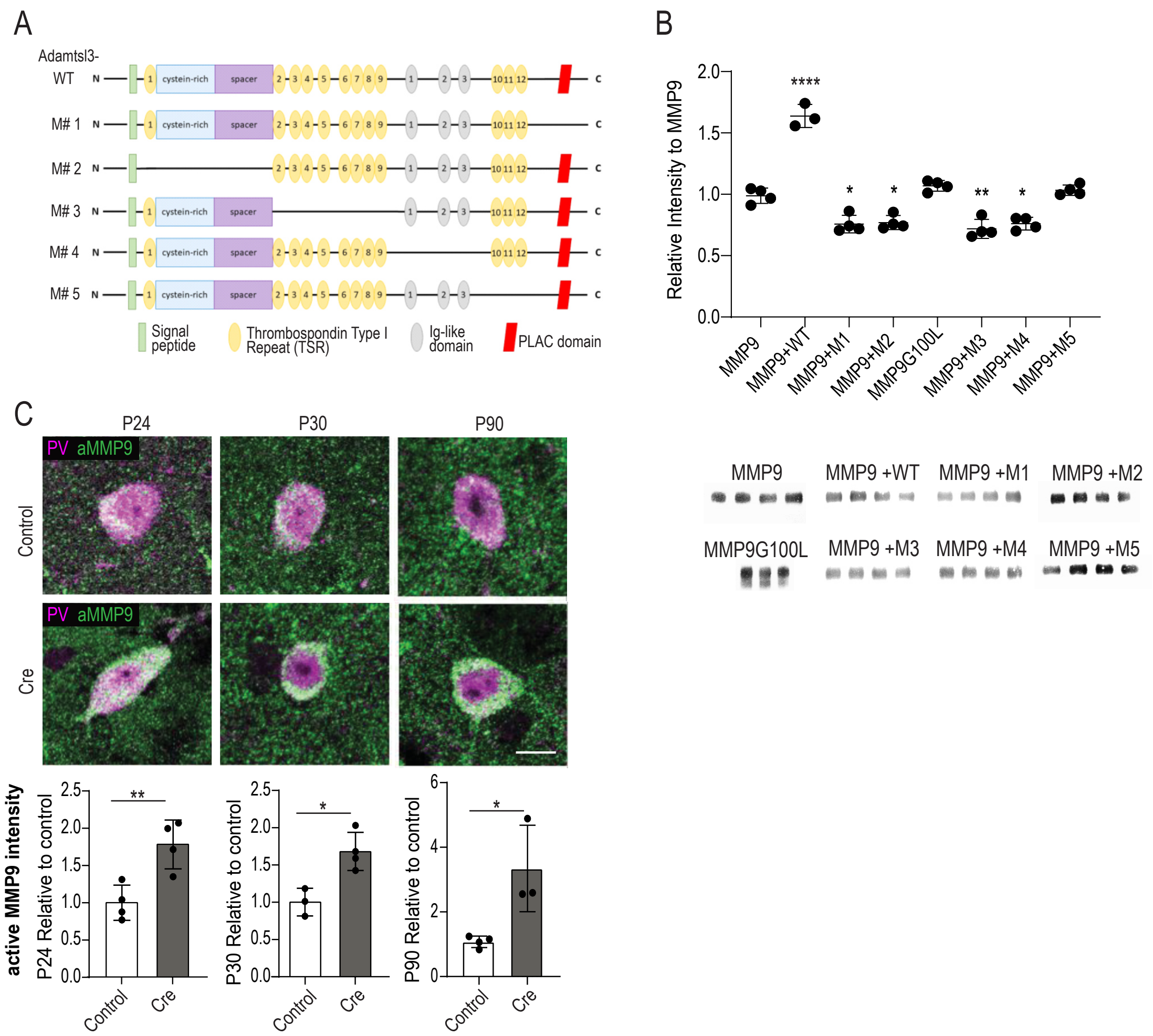

Supplementary Figure 4. Adamtsl3 regulates MMP9 activity via distinct domains.

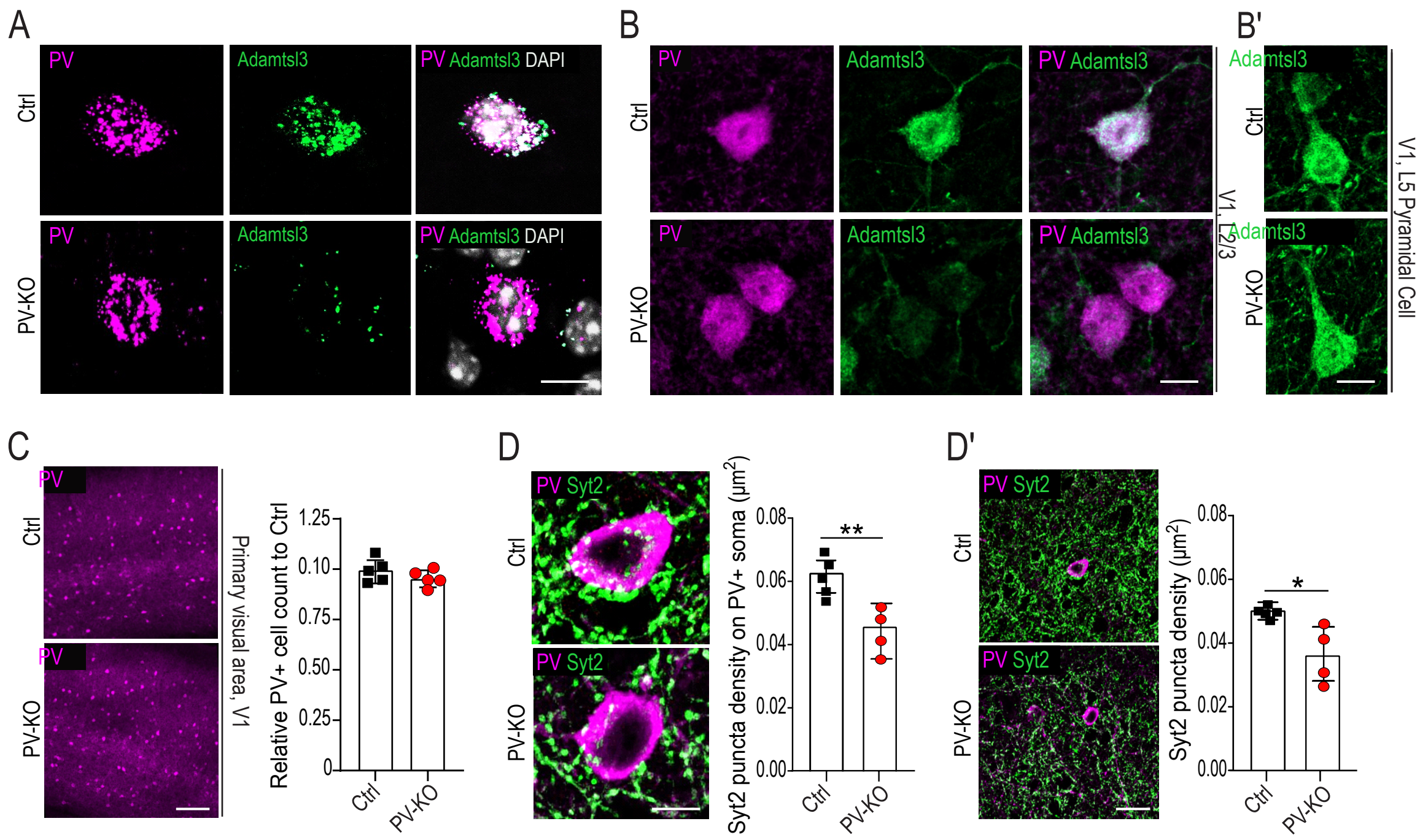

Supplementary Figure 5. PV cell-specific deletion of Adamtsl3 influences PV+ synaptic connectivity.

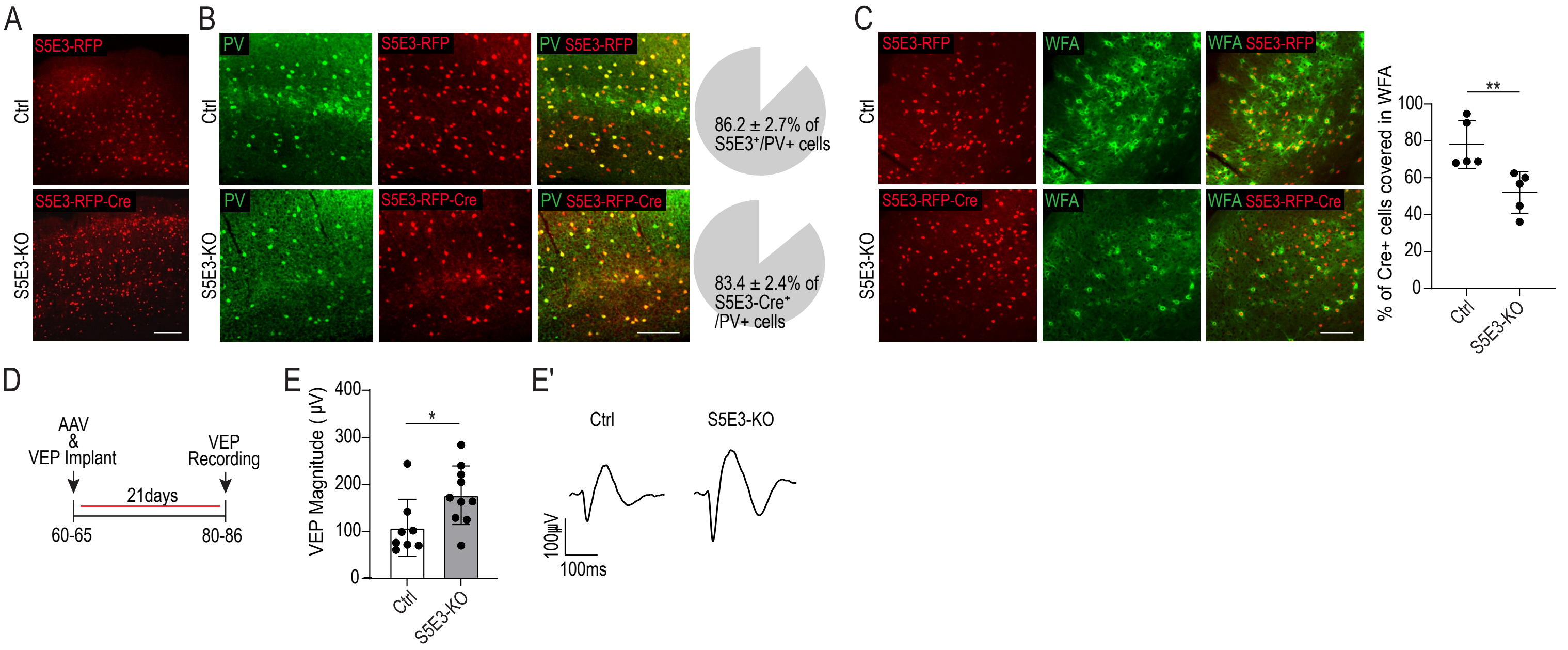

Supplementary Figure 6. Adult PV-specific Adamtsl3 deletion reduces PNNs and increases VEP magnitude.
